# Defining an EPOR- Regulated Transcriptome for Primary Progenitors, including Tnfr-sf13c as a Novel Mediator of EPO- Dependent Erythroblast Formation

**DOI:** 10.1371/journal.pone.0038530

**Published:** 2012-07-13

**Authors:** Seema Singh, Arvind Dev, Rakesh Verma, Anamika Pradeep, Pradeep Sathyanarayana, Jennifer M. Green, Aishwarya Narayanan, Don M. Wojchowski

**Affiliations:** 1 Center of Excellence in Stem Cell Biology and Regenerative Medicine Maine Medical Center Research Institute, Scarborough, Maine, United States of America; 2 Affymax, Inc., Palo Alto, California, United States of America; 3 Strand Life Sciences, Hebbal, Bangalore, India; Kyushu Institute of Technology, Japan

## Abstract

Certain concepts concerning EPO/EPOR action modes have been challenged by in vivo studies: *Bcl-x* levels are elevated in maturing erythroblasts, but not in their progenitors; truncated EPOR alleles that lack a major p85/PI3K recruitment site nonetheless promote polycythemia; and *Erk1* disruption unexpectedly bolsters erythropoiesis. To discover novel EPO/EPOR action routes, global transcriptome analyses presently are applied to interrogate EPO/EPOR effects on primary bone marrow-derived CFUe-like progenitors. Overall, 160 EPO/EPOR target transcripts were significantly modulated 2-to 21.8-fold. A unique set of EPO-regulated survival factors included *Lyl1, Gas5, Pim3*, *Pim1*, *Bim*, *Trib3* and *Serpina 3g*. EPO/EPOR-modulated cell cycle mediators included *Cdc25a*, *Btg3, Cyclin-d2*, *p27-kip1*, *Cyclin-g2* and *CyclinB1-IP-1*. EPO regulation of signal transduction factors was also interestingly complex. For example, not only *Socs3* plus *Socs2* but also *Spred2, Spred1* and *Eaf1* were EPO-induced as negative-feedback components. *Socs2*, plus five additional targets, further proved to comprise new EPOR/Jak2/Stat5 response genes (which are important for erythropoiesis during anemia). Among receptors, an atypical TNF-receptor *Tnfr-sf13c* was up-modulated >5-fold by EPO. Functionally, Tnfr-sf13c ligation proved to both promote proerythroblast survival, and substantially enhance erythroblast formation. The EPOR therefore engages a sophisticated set of transcriptome response circuits, with Tnfr-sf13c deployed as one novel positive regulator of proerythroblast formation.

## Introduction

As committed erythroid progenitors transit through a CFU-e stage, proerythroblast formation becomes dependent upon key signals transduced by EPO's cell surface receptor (EPOR). Interest in better understanding EPO effects (and EPOR action mechanisms) recently has intensified. This is based, in part, on the clinical emergence of new EPO orthologues and mimetics [1], and on EPO's ability to cytoprotect select non-hematopoietic tissues from ischemic injury [2]; to regulate select immune responses [3]; and to modulate susceptibility to diabetes [4]. Via poorly understood routes, EPO also may be associated with hypertensive and thrombolytic events [5], and as used to treat the anemia of chemotherapy may worsen the progression of certain cancers [6].

With regards to action mechanisms, the EPOR occurs pre-assembled with Jak2 kinase (as apparently paired dimer sets) [7], [8]. EPO binding conformationally alters EPOR complexes [8]. This leads to Jak2 activation, and the phosphorylation of up to eight cytoplasmic EPOR PY sites [1]. One EPOR/Jak2 signaling axis involves EPOR PY479 recruitment of p85-alpha plus p110 PI3K [9]. Disruption of p85-alpha is known to limit fetal erythropoiesis (and leads to the sustained expression of nucleated erythrocytes) [10]. Nonetheless, mutated EPOR forms that lack this PY479 PI3K docking site efficiently support erythropoiesis [11]. Fully PY-deficient EPOR forms that retain only a box-1,2 Jak2 binding domain also can support erythropoiesis at steady-state, but are markedly defective during anemia [12], [13]. A pathway that couples to EPOR PY- independent mechanisms involves a Ras/Raf/Mek/Erk axis [13]. However, candidate necessary-and-sufficient roles for Erk's have been discounted by the recent observation that erythropoiesis can be bolstered when *Erk1* is disrupted [14].

A third central EPOR signaling route involves a Jak2-plus-Stat5 axis which has been shown to be important for EPO-dependent erythropoiesis during anemia [12]. Original studies of *Stat5* disruption per se yielded disparate results for erythropoietic roles [15], [16]. Full deletion of *Stat-5a* and -*5b* loci, however, has since been shown to markedly compromise erythropoiesis [17]. Among candidate Stat5 targets, *Bcl-x* previously was proposed to comprise one important EPO/EPOR- response factor whose anti-apoptotic actions might largely explain EPO's effects [16]. Follow-up studies in primary bone marrow erythroid progenitor cells, however, have challenged this EPO/EPOR- *Bclx* connection, and instead point to roles for Bcl-xL within maturing late-stage erythroblasts [18], [19]. Together, such considerations raise important questions concerning how much is well understood about key EPO/EPOR response circuits, and effects.

Towards advancing insight into EPO/EPOR action, our laboratory recently has applied basic gene profiling approaches to initially identify select EPO-modulated targets, and for these few factors has generated basic evidence for functional significance. Examples include *Podocalyxin* as a proposed mediator of erythroblast adhesion/migration [20]; *Cyclin G2* as an EPO/EPOR- repressed cell cycle inhibitor [21]; and *Serpina-3g* as an EPO- induced candidate erythropoietic factor [19]. To broaden insight into EPO action mechanisms, we presently report on global transcript response events that EPO regulates within primary bone marrow CFUe- like progenitors. Attention is first given to candidate mediators of EPO's effects on response genes. Subsequent analyses address functional sets of EPO/EPOR targets which proved to include unique sets as regulators of proerythroblast survival, cell cycle progression, signal transduction, negative-feedback factors, and cytokines plus receptors. Within each functional sub-set (including delineated EPOR/JAK2/STAT5 targets), specific EPO/EPOR- modulated factors are described. Among cytokines-plus-receptors, one prime EPO-EPOR induced target proved to be a pro-survival TNF receptor, *Tnfr-sf13c* [22], [23]. As engaged in lymphoid cells by its BAFF ligand (B-cell activating factor TNF family), Tnfr-sf13c is essential for lymphoid progenitor cell survival and B-cell formation [23]. Within primary erythroid progenitors, Tnfr-sf13c (as induced by EPO) is now shown to enhance proerythroblast survival, and in addition, to promote the formation of late-stage Ter119^pos^ erythroblasts. Overall findings are discussed in the contexts of unique response circuits that the EPOR regulates within primary bone marrow progenitors to sustain the balanced production of red blood cells at steady-state, and over a dynamic range of rates during anemia.

## Materials and Methods

### Mouse models

Unless otherwise indicated, C57BL/6 mice (Jackson Laboratory) were used at age 8–14 weeks as a source of bone marrow erythroid progenitor cells (EPC's). Mice harboring the knocked-in minimal EPOR alleles EPOR-H, and EPOR-HM were as characterized by Menon et al [12]. All protocols and procedures were approved by the IACUC of the Maine Medical Center Research Institute (Protocol number 0911).

### Erythroid progenitor cell culture and isolation

Bone marrow cells were prepared from femurs and tibiae as previously described [19], [20], [21], [24]. EPC's were then expanded in SP34ex medium supplemented with 2.5 U/mL EPO, 100ng/mL mSCF, 1uM dexamethasone, 1 uM beta-estradiol, 75 ug/mL holo-transferrin, 0.1 mM 2-mercaptoethanol, 1.5 mM glutamine, 0.5% BSA (Stem Cell Technologies) [24]. At day 3.5 of culture, CFUe-like stage E1 progenitors, stage E2 proerythroblasts, and stage E3 erythroblasts were isolated. Here, optimized multi-step MACS-based procedures were employed as recently detailed [24], and yielded purities of ≥99.9%.

### Flow cytometry

In analyses of stage E1, E2 and/or E3 cells (and per 200 uL assay), 10^6^ cells were incubated (15 minutes, 4°C) with 5 ug of rat IgG in PBS, 0.5% BSA (0.2 mL assay volumes). PE-Ter119, FITC anti-CD71, and APC-anti-Kit antibodies (1 ug each, BD Biosciences) then were added (30 minutes, 4°C). Washed cells were analyzed by flow cytometry (BD FACScalibur, Cell Quest software). In assays of cell survival, Annexin-V or YoPro3 were used as recently detailed [19], [24]. Viable cell numbers and frequencies were assayed by Vicell assays. In all experiments, equivalent numbers of gated events were analyzed.

### Gene profiling analyses

In analyses of EPO/EPOR response genes, purified stage E1 CFUe- like progenitors were cultured in the absence of EPO for 5.5 hours in HEPES buffered IMDM supplemented with 10 µg/mL transferrin, 15 ng/mL insulin, 0.1 mM 2-mercaptoethanol, 0.5% BSA, 1.5mM glutamine. Cells were then exposed to EPO (4 U/mL) or carrier (HSA, PBS). At 90 minutes of exposure, cells (from four independent replicates, each plus- and minus- EPO) were lysed directly in Trizol reagent. RNA was then isolated and used to prepare biotinylated probes for array hybridizations. Gene profiling utilized Affymetrix 430 2.0 arrays, GeneChip 3000 scanning and initial GCOS software (Affymetrix) analyses of hybridization signals. Subsequent bioinformatics analyses utilized GeneSpring GX 11.0. Microarray data were assessed for background signals, normalized, and probe-summarized using a GC-RMA algorithm. Significantly expressed genes were identified using a Benjamini-Hochberg FDR filter. For EPO modulated genes, a p-value cut-off of 0.05 was used. K-means clustering utilized a Euclidean distance matrix. For significance testing between [−] EPO vs [+] EPO samples, Student's T-testing was used (single tailed). Analyses of transcription factor binding sites represented within K-means clusters #1 – #4 of EPO- modulated genes was via DiRE [25], [26]. To further estimate the significance of STAT elements as represented among Cluster #4 EPO-response genes, TransFac was applied (−950 to +50 algorithm).

### RT-PCR

Reverse transcription (standard 1 ug of RNA per reaction) was with Superscript III (and included pre-treatment with DNAse I) (Invitrogen). Quantiative PCR utilized Sybr-green reagents and MyIQ single color real-time PCR detection system (BioRAD). Primer pairs were from SuperArray, and are defined in supplemental materials (Table S1).

## Results

### Defining an EPO/EPOR- modulated transcriptome within primary CFUe- like progenitors

**Figure 1:**
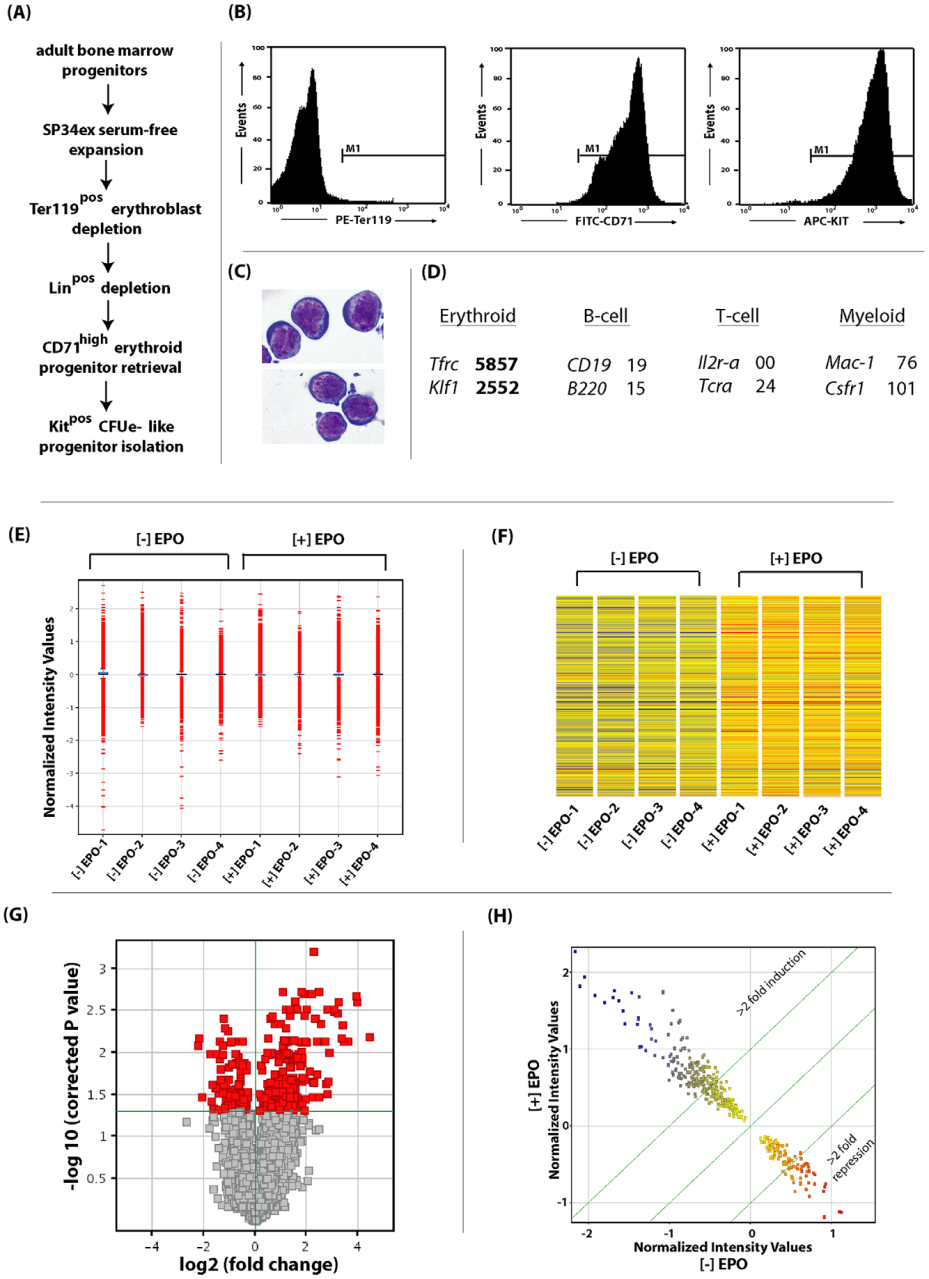
Defining the EPO/EPOR- regulated transcriptome in primary bone marrow CFUe-like erythroid progenitor cells. **A**] Short term expansion of primary bone marrow erythroid progenitors, and isolation of a CFUe- like cohort – Approaches employed for the expansion and purification of Kit^pos^CD71^high^Ter119^neg^ stage E1 progenitor cells are outlined. **B, C**] For isolated stage E1 cells, flow cytometric features are illustrated, together with representative May-Grumswald cytospin preparations. **D**] For CFUe-like progenitors (stage E1) purity also was assessed based on initial overall transcriptome profiling (n = 3 per stage), with relative mean expression intensities illustrated for select erythroid (*Tfrc*, *Klf1*), lymphoid (*CD19*, *B220*, *IL2r-a*, *Tcr-a*) and myeloid *(Mac1, Csfr1*) marker transcripts. **E**] Distributions and variance of overall hybridization signal intensities among control and EPO- challenged treatment groups. **F**] Heat-map signatures of expressed transcripts (for all independent quadruplicate samples) for [−] EPO vs [+] EPO exposed CFUe-like stage E1 progenitors. Here, a fold cut-off of >1 was used, and signal strengths for 365 transcripts (probe sets) are illustrated. **G**] In this volcano plot, the dividing line (and vertical color-code) denote significant p-values (0.05 cut-off) for EPO- modulated transcripts (−log_10_ ordinate scale). The x-axis displays transcripts based on their fold-change due to EPO (log_2_ scale). **H**] Overall scatter plot of significantly modulated EPO/EPOR- response genes.

During definitive erythropoiesis, development beyond the CFU-e stage fails due to disrupted expression of *Epo,* or the *EpoR* [27]. Such CFUe- like cells, including those generated using an optimized serum-free ex vivo system for murine bone marrow erythroblast development [20], [21], [22], [24], exhibit sharp dependency on EPO for their growth and survival. Present global transcriptome analyses therefore focused on this developmental cohort of Kit^pos^CD71^high^Ter119^neg^ progenitors (termed “stage E1”). Figure 1A defines approaches employed for the short-term expansion, and isolation of these primary progenitors. For stage E1 cells, the high purity routinely obtained is illustrated via flow cytometric analyses of Kit^pos^CD71^high^Ter119^neg^ cells (Figure 1B). This was further confirmed through transcriptome-based analyses of erythroid and possibly contaminating B-cell, T-cell and myeloid cell markers. May-Grumwald cytospins of stage E1 cells also are shown (Figures 1C, 1D).

Pilot experiments next served to assess time-courses for EPO-induction of four known response genes as *Cis*, *Pim1, Socs3* and *Podocalyxin*. As analyzed at 10, 30, 90 and 270 minute intervals, near maximal induction of these target genes was achieved by 90 minutes (data not shown). With regards to ligand concentration, EPO levels during anemia can increase several hundred-fold [1] (and EPO at 3U/mL is required to support stress BFU-e development, for example) [28]. An EPO challenge dose of 4U/mL therefore was used in profiling experiments. Using the above defined conditions, purified CFUe-like cells next were interrogated for EPO/EPOR modulated events using Affymetrix arrays. Specifically, quadruplicate independent samples were interrogated for EPO- challenged stage E1 cells versus unchallenged controls. In these (and all profiling experiments presented below), all samples readily passed all quality control parameters, and signal variance was distributed similarly among replicates (Figure 1E). Figures 1F and 1G illustrate heat-maps for each sample, together with a volcano plot of p-value distributions. When analyzed via GC-RMA algorithms (p≤0.05) and unpaired Student's T-testing), 196 arrayed genes proved to be significantly EPO- modulated (Figure 1H). Among these target genes, 157 were up-modulated, and 39 were down-modulated (with mean fold- modulations of 3.6 fold, and 2.6 fold, respectively).

**Figure 2:**
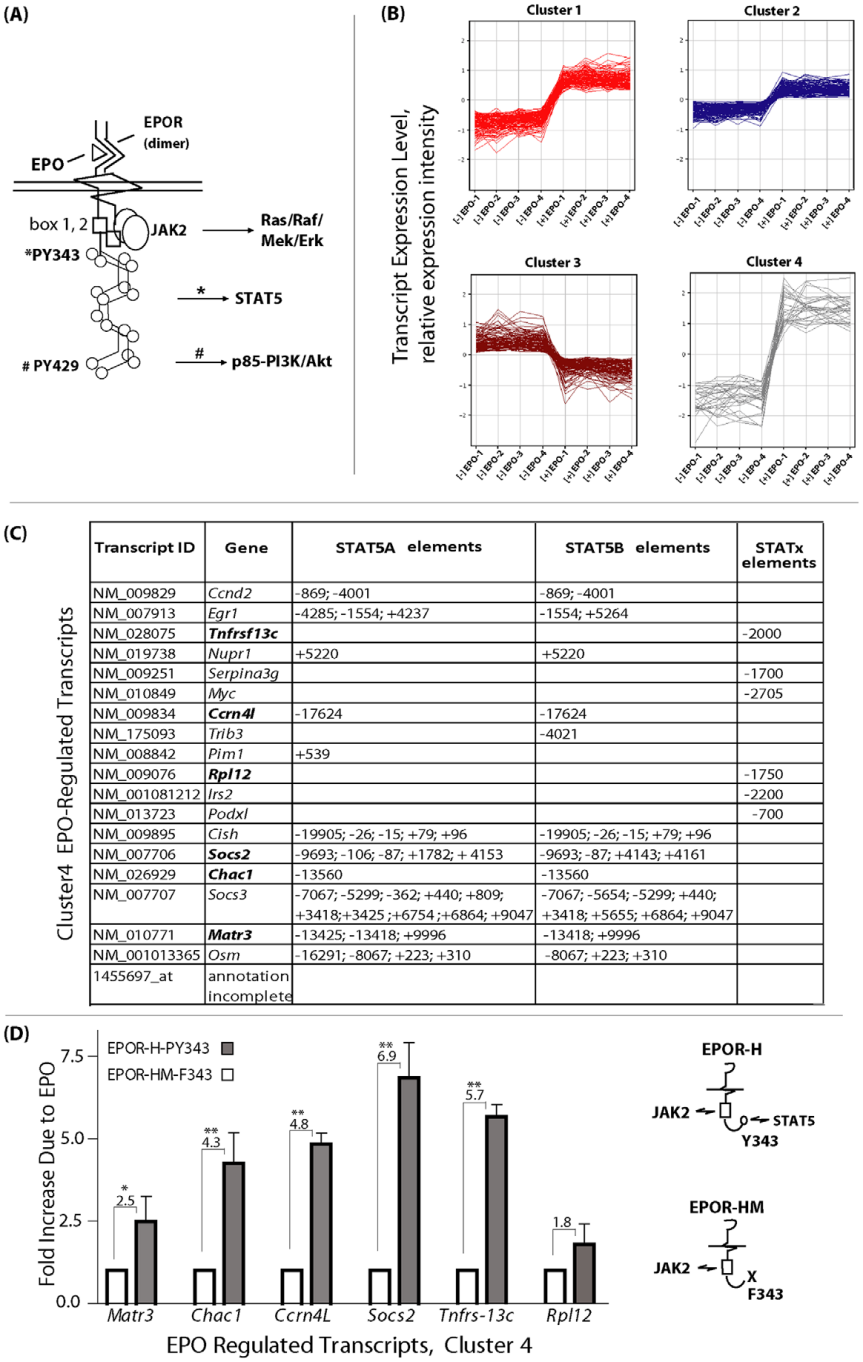
K-Means clustering of EPO/EPOR- modulated gene sets, and identification of novel EPOR/JAK2/STAT5 targets. **A**] The EPOR is diagrammed, together with subdomains that engage canonical Ras/Raf/Mek/Erk, Stat5, and PI3K/Akt response pathways. **B**] K-means clustering of four predominantly patterned EPO/EPOR– response gene subsets **C**] Proposed nature of “cluster-4” EPO- response genes as EPOR-PY343/Stat5 targets. **D**] Quantitative RT-PCR analyses of novel “cluster-4” EPO/EPOR response genes for isolated CFUe- like progenitors expressing wild-type, PY site-deficient, or PY343 Stat5- coupled EPOR alleles (wt, EPOR-HM, EPOR-H). In RT-PCR, normalization was to *beta-actin*. Values are means of duplicate assays.

A consideration of core structure-function features of the EPOR (Figure 2A) prompted an unsupervised clustering approach to sub-categorize EPO/EPOR- response genes. K-means clustering of modulated transcripts resolved four major patterned response sets: 1- induced, 2- moderately induced, 3- repressed, and 4- markedly induced (Figure 2B). This analysis suggested that possible groupings of transcription factor sets may differentially modulate select clusters of EPO/EPOR target genes. In this context, cluster-4 was considered in further detail. Notably, based initially on in silico analyses, essentially all EPO- modulated targets in this subset proved to comprise either known, or predicted Stat5 (or Stat X) targets (Figure 2C). This included six new candidate Stat5 target genes within CFUe- like progenitors as *Matr3*, *Chac1*, *Ccrn4l*, *Socs2*, *Tnfr-sf13c* and *Rpl12*. To critically assess the candidate nature of these targets as EPO/EPOR/Stat5 response genes, independent quantitative RT-PCR analyses were performed using CFUe-like progenitors isolated from mice harboring knocked-in EPOR-HM and EPOR-H alleles. The minimal EPOR form EPOR-HM is coupled to JAK2 but lacks all cytoplasmic PY sites, while EPOR-H retains a single PY343 site for Stat5 recruitment [12]. CFUe-like stage-E1 progenitors from bone marrow of EPOR-HM and EPOR-H mice were expanded, purified and cultured without EPO for 5.5 hours. Cells then were exposed to EPO (+/−4U/mL). At 90 minutes RNA was isolated, and reverse-transcribed. Quantitative PCR analyses provided independent evidence that each of the above novel cluster-4 EPO response factors represent an EPOR-PY343-Stat5 target (Figure 2D). DiRE analyses of cluster-4 also indicated ∼33% representation of STAT sites among ten top- occurring transcription factor binding sites (and scored 2^nd^ of 10 in indicated importance) (Figure S1). As points of comparison, DiRE was also applied to analyze predominant representations of transcription factor elements for EPO- response genes within clusters #1, #2 and #3. Within EPO- response gene clusters #1 and #2, STAT binding sites were not enriched (among top-20 enriched motifs). In addition, no known STAT5- induced genes occurred among cluster #1 EPO targets, and within cluster #2 only *Cyclin-d2* was represented (and only for a single probe set signal). To inform further, all EPO- modulated genes within each cluster are listed in Table S2 (together with DiRE- predicted transcription factor binding sites). In contrast, within cluster #4 *Cyclin-d2* was represented for five probe sets, and *T-cell receptor-gamma* for three probe sets (for example), data not shown. In addition, cluster #4 contained a number of EPO- modulated genes that previously have been implicated as STAT5 targets (e.g., *Socs3*, *Cis*, *Pim1*, *Podocalyxin*). STAT5a elements, however, were enriched in DiRE analyses of cluster #3 (although at a lower scoring) (note: DiRE searches and scores full gene loci for conserved, weighed transcription factor binding sites. Positive scores are reported based on numbers of sites with summation for non-coding region sites. DiRE also scores the association of individual transcription factors with the biological function shared by groups of input genes [25], [26]). Cluster #3 includes transcripts that are down-modulated by EPO. In support of the apparent enrichment of STAT5a sites, inspection of the gene list for cluster #3 (see Table S2) revealed *Cyclin G2*, *Trb2*, and *Klf3*, each of which previously has been implicated to be subject to repression by STAT5 [19], [21], [29]. By speculation, STAT5a therefore might be more involved in repression within an EPO- response context as contrasted to STAT5b.

### Functional classes of EPO/EPOR targets

**Figure 3:**
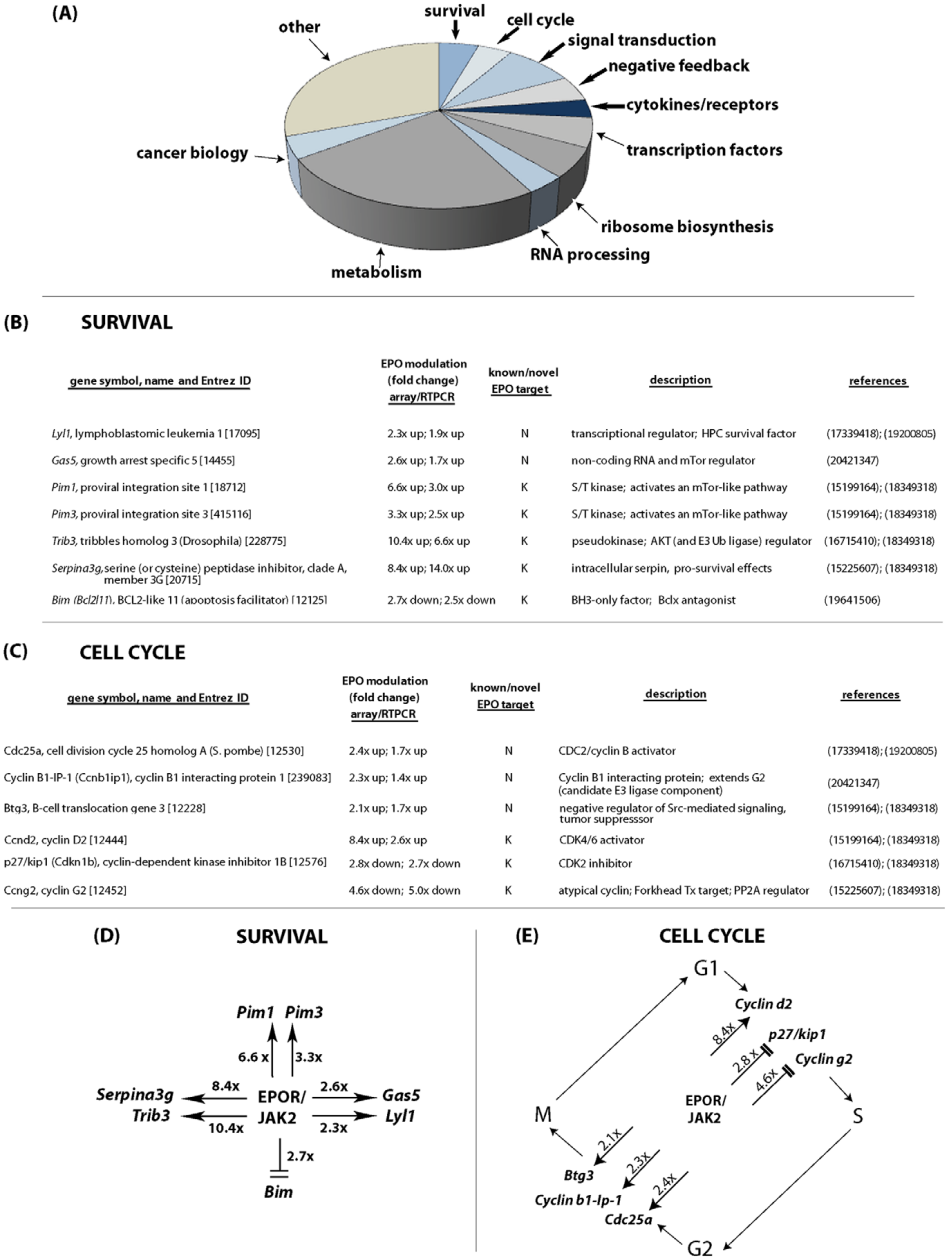
Subsets of functional targets within the EPO/EPOR response transcriptome, including survival and cell cycle factors. **A**] Frequencies of EPO/EPOR- modulated targets are defined within eleven functional sub-categories. **B, D**] Among survival factors, EPOR ligation significantly modulated seven prime factors (6 induced, plus *Bim* repressed). **C, E**] Cell cycle factors as modulated by EPO/EPOR ligation included three involved in phase- G1 progression; and three that regulate phase G2.

In keeping with broadening concepts for EPO's effects [1], [2], [3], [4], [5], [6], functional sub-sets of EPO/EPOR- modulated targets included survival factors, cell cycle regulators, signal transduction factors, negative feedback factors and a select set of cytokines plus receptors (as defined in some detail below). In addition, a substantial number of EPO/EPOR response factors sorted to functional categories of transcriptional regulators, cancer biology- associated factors, ribosome biosynthesis regulators, RNA processing factors and metabolic factors. These overall functional sub-sets of EPO/EPOR- modulated factors are outlined in Figure 3A. The latter categories of EPO/EPOR targets are of significant interest, and therefore are further defined in Tables S3, S4, S5, S6, S7, S8.

One prime role ascribed to EPO/EPOR signaling involves anti-apoptotic effects. In highly EPO-dependent CFUe-like stage E1 erythroid progenitors, seven cell survival factors proved to be clear EPO/EPOR target genes (Figure 3B). *Pim1* S/T kinase has previously been described. *Pim3* (like *Pim1*) acts via an mTOR-like pathway [30]. In parallel, *Gas5*, a non-coding RNA that supports mTor expression also was induced (2.6-fold). *Lyl1* also comprised a novel EPO/EPOR target, and recently has been shown to act (as a transcriptional regulator) to promote hematopoietic progenitor cell survival [31]. Among Bcl2-related factors, only *Bim* was EPO/EPOR regulated (2.7-fold repression of this BH3-only, proapoptotic factor). Finally (and as reported recently) [19] the intracellular serpin *Serpina-3g* and the pseudokinase *Trb3* each were strongly EPO-induced. Each has potential anti-apoptotic activities, and each comprises a singular EPO-modulated orthologue within multi-member families (e.g., *Tribs-1, -2 and -3*) (data not shown). Notably, modulation of the above EPO/EPOR-response genes (Figure 3D) also was validated by quantitative RT-PCR (Figure 3B, and Figure S2).

Select cell cycle regulators recently have been indicated to also be subject to EPO- modulation. Specifically, the inhibitory cyclin, *Cyclin-G2* has been described as a target for EPO repression, and *Cyclin-D2* as a target for EPO-induction (each, in part, via an EPOR/Jak2/Stat5 axis) [17], [19]. Beyond *Cyclins G2* and *D2*, global transcriptome analyses revealed four additional EPO-modulated cell cycle regulators (Figure 3C). These first included *Btg3* as an E2F-1 regulator [32] (and novel EPO/EPOR target). Also modulated were *p27/Kip1* as a repressed target (and CDK2 inhibitor) together with two regulators of phase G2 progression as *Cdc25a* and a Cyclin B interacting protein, *Cyclin B-IP1*. EPO therefore appears to engage a select collection of cell cycle regulators which overall are proposed to promote G1 and G2 phase progression within rapidly dividing CFUe-like progenitors. For the above cell cycle regulators (Figures 3C, 3E) EPO/EPOR modulation also was confirmed by RT-PCR (Figure 3C, and Figure S2).

### EPO/EPOR modulation of cytokines/receptors, negative-feedback factors, and signal transduction factors

**Figure 4:**
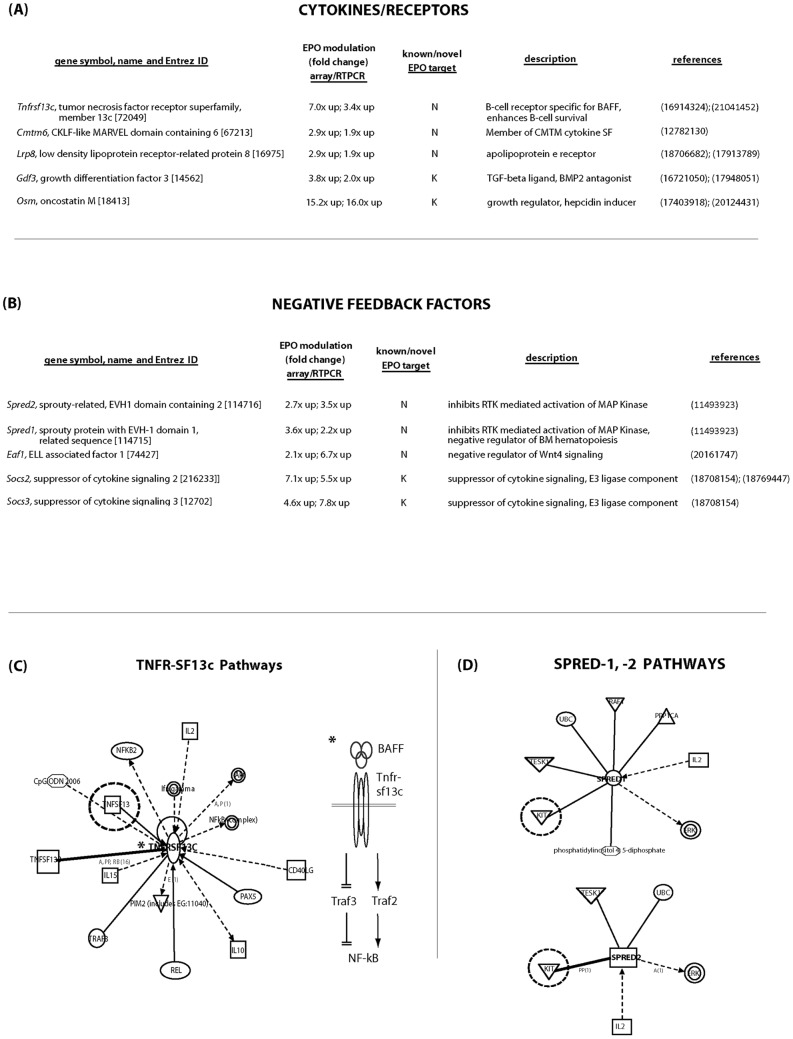
EPO/EPOR – modulation of cytokines plus receptors, and negative feedback factors. **A**] Cytokines/ receptors modulated due to EPOR ligation included *Tnfr-sf13c* (BAFF receptor), *Cmtm6* (transmembrane chemokine receptor like superfamily-6), *Lrp8* (cholesterol co-receptor), *Gdf3* (a BMP antagonist), and *Oncostatin-M* (a new indirect mediator of iron transport). **B**] Among negative-feedback factors, EPOR ligation up modulated five prime factors as *Spred2, Spred1, Edf1, Socs2,* and *Socs3*. **C**] For Tnfr-sf13c, associated factors and pathways are outlined (based on Ingenuity algorithms). **D**] For Spred-1 and -2, associated factors and pathways are outlined.

Next, with regards to an extended category of EPO/EPOR targets as additional signal transduction factors (STF's), global analyses revealed 22 such factors to be significantly modulated. Two STF sub-categories were cytokines plus receptors, and negative feedback factors (Figure 4) (guiding references also are cited in Figure 4 as PMIDs) (also see Figure S3). Among receptors, a TNF receptor, *Tnfr-sc13c,* proved to be most strongly induced (3.4 to 7.0 – fold up-modulated due to EPO). Tnfr-sc13c binds BAFF ligand, and as studied to date in B-cells [22], [23] is an atypical TNF-R which exerts pro-survival effects (Figures 4A and 4C). This factor therefore is the subject of extended functional erythropoietic studies (see following Results sub-section). *Lrp8*, a receptor for apolipoprotein-E ligands also was induced 1.9 – to 2.9 – fold. Interestingly, roles for Lrp8 in platelet function and neural cell migration recenty have been described. In stage-E1 EPC's, EPO also induced the expression of three cytokines: *Cmtm6*, *Gdf3* and *Oncostatin-m*. Cmtm6 is a chemokine-like factor superfamily member with presently unknown function, while Gdf3 can act as a TGF/BMP antagonist (and may therefore counter inhibitory effects of TGFbeta on erythropoiesis). *Oncostatin-m* is a known EPO response gene, but recently has been shown to induce hepatocyte hepcidin expression, and to consequently decrease serum iron levels [33].

EPO/EPOR STFs also included five negative feedback factors (Figures 4B, 4D) (guiding references are included as PMIDs) (also see Figure S3). Two are suppressors of cytokine signaling, while three are novel EPO targets as *Spred2*, *Spred1* and *Eaf1*. SOCS factors act as E3 ubiquitin ligases, and exert feedback effects on activated EPOR/Jak2 complexes [34]. The present results specifically implicate not only *Socs-3* but also *Socs-2* in this feedback loop. Spreds are adaptor proteins that have been characterized as negative feedback factors in RTK systems [35], but not in the context of Janus kinase- coupled receptor modulation. Finally, Eaf1 can act as an inhibitor of Wnt signaling. For twelve additional EPO/EPOR modulated STF's, these are described in supplemental text associated with Table 1 (which includes references as PMID's). As defined in Table 1, these include.

**Table 1:** EPO/EPOR-modulated signal transduction factors.

gene symbol, name and Entrez ID	EPO modulation (fold change) array/RTPCR	known /novel EPO target	Description	references
*Pik3cb*, phosphatidylinositol 3-kinase catalytic beta subunit [74769]	2.6× up	N	p110beta catalytic subunit of PI3K	(18594509); (19815050)
*Mobkl1a*, MOB1, Mps One Binder kinase activator-like 1A [68473]	2.2× up	N	NDR kinase regulator	(20624913); (18328708)
*Prkcq*, protein kinase C-theta [18761]	2.3× up	N	PKC theta S/T kinase	(20592275); (12670396)
*Gnb2l1*, G protein, beta polypeptide 2 like 1 [14694]	3.0× up	N	G-protein beta 2-like-1 (anchor for PKC)	(19767770); (20093473)
*Gab2*, growth factor receptor bound protein 2-associated protein 2 [14389]	2.7× up	N	Grb2 and SHP2 docking protein	(14662016); (20161778); (17374739)
*Irs2*, insulin receptor substrate 2 [384783]	7.4× up	K	insulin and IL4PY- regulated docking protein	(19109238); (12506011)
*Erbb2ip*, Erbb2 interacting protein [59079]	3.1× down	N	Erbin, RTK and TGF-R modulator	(17591701); (20887249)
*Tirap*, TIR domain-containing adaptor protein [117149]	2.4× up	N	IL1 and Toll receptor adaptor	(12447442); (19898489)
*Gnl3*, guanine nucleotide binding protein-like 3 (nucleolar) [30877]	2.3× up	N	nuclear receptor negative co-regulator	(20703089); (17623774)
*Pnrc1*, proline-rich nuclear receptor coactivator 1 [108767]	2.3× up	N	nuclear receptor positive co-regulator	(10894149); (20023006)
*Nol8*, nucleolar protein 8 [70930]	3.1× up	N	GTP binding protein – binding protein	(15132771); (14660641)
Plek2, plecstrin 2 [27260]	3.9× up	N	Pleckstrin2, PIP-regulated cytoskeletal factor	(17658464); (17008542)

Twelve additional signal transduction factors (STFs) were defined as significantly EPO/EPOR- modulated targets: two are kinases, as *PI3K p110beta* and *PKC theta*; others include *Rack1/Gnb2l1*, *Erbin/Erbb2ip* (an RTK modulator), two docking proteins (*Gab2, Irs2*), and *Pleckstrin2*. References are cited by PMID number.

Among twelve remaining EPO/EPOR modulated STF's (Table 1, including references as PMID's), one has been reported previously as insulin receptor substrate-2 (*Irs2*), a docking protein also utilized by the IL4R. Eleven represent novel EPO- response factors. One similarly is a docking protein, *Gab2*, while two are kinases as PI3K's catalytic beta subunit (*Pik3cb*), and PKC-theta (*Prkcq*) (an NFKb regulator). Another, MOB1 (*Mobkl1a*) is a preferred substrate of Mst1/2 Ste20- like kinases. Three are G- (or G-like) proteins (or binding proteins) as *Gnb2l1*, *Gnl3* and *Nol8*; and three are regulating co-factors for RTKs, Toll receptors, and nuclear receptors as *Erbin/Erbb2ip*, *Tirap*, and *Pnrc1*, respectively. Finally, one EPO/EPOR response factor is a regulator of cytoskeleton restructuring as Pleckstrin-2 (*Plek2*) (3.9-fold induction). Thus, EPO also modulates the expression of intriguingly diverse sub-sets of novel STF's with functions that will be of significant interest to further delineate (and network).

Pik-3cb, Mobkl1a, Prkcq, Gnb2l1, Gab2, Irs2, Erbb2ip, Tirap, Gnl3, Pnrc1, Nol8, Plek2.

### Functional roles for Tnfr-sf13c during erythropoiesis

**Figure 5:**
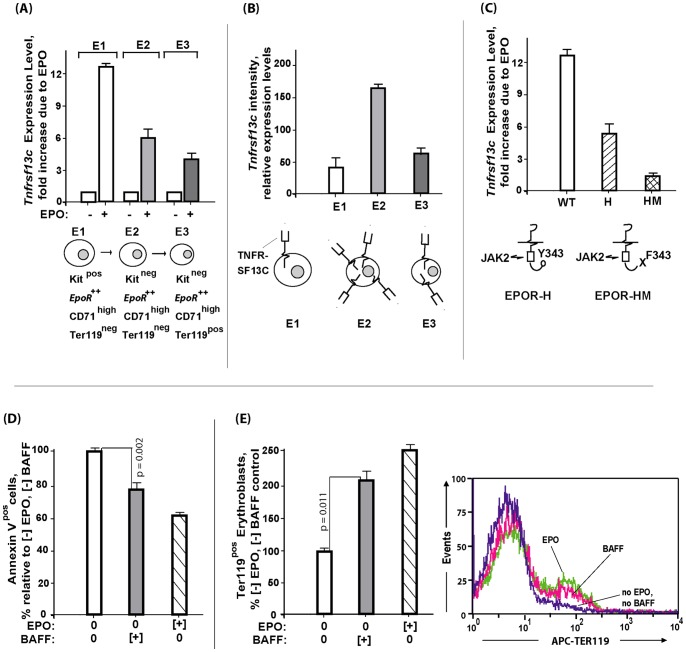
Tnfr-sf13c expression during EPO-dependent (pro)erythroblast development, and erythropoietic effects of BAFF. **A**] EPO efficiently induces *Tnfr-sf13c* expression in CFUe-like progenitors, proerythroblasts, and Ter119^pos^ erythroblasts (stages E-1, -2, and -3, respectively)- Stage E1, E2, and E3 EPC's were isolated; cultured for 5.5 hours in the absence of EPO; and then EPO-challenged frequencies of Ter119^pos^ stage E3 erythroblasts were determined (by flow cytometry) (means +/− SE, n = 4). for 90 minutes (4U/mL). RNA was isolated, reverse-transcribed and used in quantitative RT-PCR analyses. **B**] Tnfr-sf13c expression peaks within stage-E2 erythroblasts. *Tnfr-sf13c* levels also were determined within stage E1, E2, and E3 EPC's directly upon isolation. **C**] EPO/EPOR- induction of Tnfr-sf13c depends, in part, upon Stat5 engagement. Stage E1 bone marrow EPC's were expanded and isolated from wild-type mice, and mice harboring EPOR-HM or EPOR-H alleles. For each, EPO induction of *Tnfr-sf13c* expression was then assayed (by RT-PCR) (mean +/− SE, n = 3 per group). **D**] BAFF- dependent inhibition of apoptosis among primary bone marrow proerythroblasts. EPCs were expanded from wild-type bone marrow. Stage E1 plus E2 cells were then isolated, cultured for 5.5 hours without EPO, exposed to EPO for 2 hours, and then washed thrice. Cells then were cultured for 15 hours in the presence (or absence) of BAFF ligand (1.2 ug/mL) or EPO (0.06 U/mL). Levels of annexin v- positive cells then were determined (via flow cytometry) (means +/− SE, n = 4). **E**] BAFF ligation of Tnfr-sf13c substantially promotes the formation of maturing Ter119^pos^ erythroblasts. Ter119^neg^ EPC's were expanded and isolated. Cells were then cultured in the presence, or absence of BAFF ligand (1.2 ug/mL) or EPO (0.06 U/mL) and at 15 hours, frequencies of Ter119^pos^ erythroblasts were determined (by flow cytometry). Graphed values are means +/− SE.

**Figure 6:**
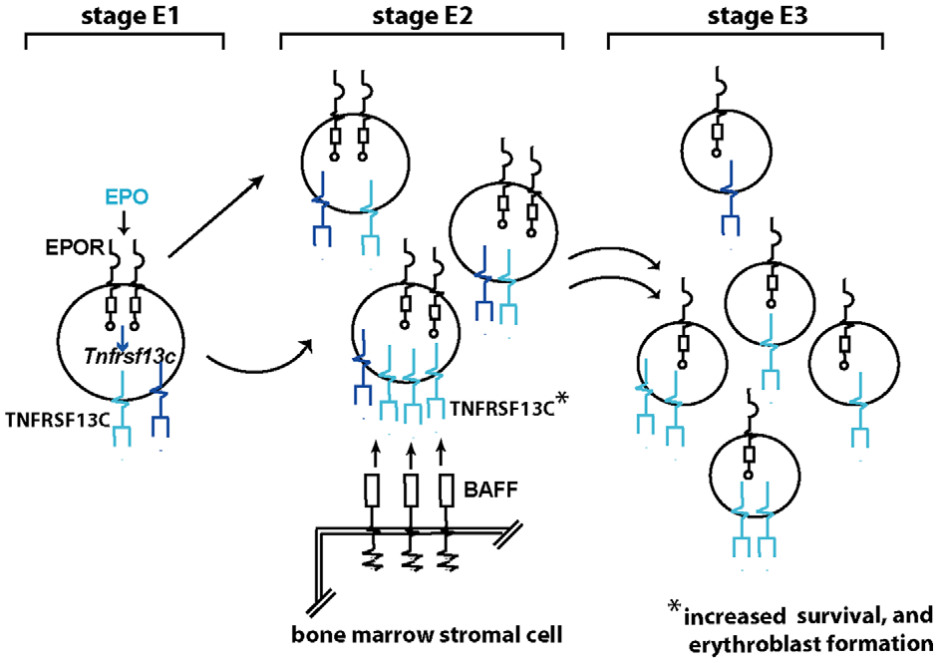
Model for EPO\EPOR deployment of Tnfr-sf13c as an agent for proerythroblast survival, and erythroblast formation. In stage E1 EPC's and stage E2 pro-erythroblasts, elevated EPO levels heighten Tnfr-sf13c expression. Tnfr-sf13c ligation via stromal cell Baff then results in heightened survival of pro-erythroblasts, and increased formation of Ter119^pos^ stage E3 erythroblasts.

Specific roles for Tnfr-sf13c during erythropoiesis next were assessed. EPO- induction in stage E1, E2 and E3 cells first was investigated, and confirmed at each stage (Figure 5A). Steady-state expression also was assayed, and proved to peak in stage E2 proerythroblasts (Figure 5B). The dependency of EPO's induction of *Tnfr-sf13c* on Stat5 also was confirmed in independent analyses using stage E1 progenitors expressing wt-EPO, EPOR-H or EPOR-HM alleles (Figure 5C). In functional experiments, primary bone marrow (pro)erythroblasts first were prepared essentially as described above for EPO-challenge experiments (including a second depletion of residual Ter119^pos^ cells). Tnfr-sf13c expression then was induced by exposure to EPO (2U/mL) for 4 hours. Cells were washed thrice, and replated in the presence or BAFF (as Tnfr-sf13c's ligand). In parallel, cells were exposed to EPO (also included were no-BAFF, and no-EPO controls). At a subsequent 15 hour time point, possible effects on survival and/or Ter119^pos^ erythroblast formation were assessed (by flow cytometry). Notably, BAFF ligand proved first to cytoprotect erythroid progenitors with an efficiency approaching that of EPO (ie, 60% of survival effects exerted by EPO) (Figure 5D). (For example primary data, also see Figure S4). In addition, BAFF (in the absence of EPO) also proved to clearly support the formation of Ter119^pos^ erythroblasts (again, with substantial efficiency) (Figure 5E). In colony-forming assays, BAFF also moderately enhanced CFUe formation (170.5+/−12.3 per 1×10^5^ BM cells with 0.5 µg/mL BAFF vs. 143.3+/−13.9 with EPO alone, 1 U/mL) (p = 0.03, Student's T-test for mean values). CFUe formation may be only modestly affected by BAFF-ligand based on apparent actions at a post-CFUe (pro)erythroblast stage. These experiments employing primary bone marrow erythroblasts provide direct evidence for BAFF-ligated Tnfr-sf13c in promoting erythroblast development, and therefore the actions of EPO. This especially applies to developing proerythroblasts with heightened Tnfr-sf13c levels (see schematic model, Figure 6). In related analyses, NCBI Geo databases were used to more broadly consider *Tnfrsf13c* expression profiles. General comparisons to receptors such as EGF-R and IFNa-R indicated relatively narrow expression distributions for *Tnfrsf13c* (121,615 and 45,101 positive profiles for EGF+R and FGFR vs 822 for Tnfrsf13c). Factors indicated to regulate Tnfrsf13c also were limited. Cytokines included SDF1, IL4 and IL2 while transcription factors included STAT5b, Zfp3L2 and Pu1 (see Table S9). For each of eight example studies, basic details on mean fold-modulation and experiment design also are provided. (For additional information, please consult the indexed NCBI Geo GDS data sets).

## Discussion

Since the cloning of EPO and its receptor, the EPOR system has served well as a paradigm for HGF-R action [1]. Much of our understanding of EPO/EPOR effects, however, depends upon ectopic over-expression in cell line models (which often have been non-erythroid, or non-hematopoietic). In addition, and based on available reagents, attention has been paid predominantly to canonical pathways and signal transduction factors. For the present studies, a prime goal was to employ a transcriptome profiling approach, together with a primary bone marrow CFUe- like target cell population, to better reveal events (initially at a transcript level) that EPO elicits in a bona fide target cell population. Further impetus was provided by unique advances in the short-term expansion and purification of uniform cohorts of bone marrow-derived EPC's (as recently detailed by Dev et al) [24]. Also, pilot transcriptional analyses in various cell line models proved to yield partially overlapping but otherwise disparate profiles among EPO response genes (eg, differentially regulated orthologues of *Pim*, *Socs*, *Spry, Spred* and *Bcl2* factors; no modulation of *PodxL*) (data not shown). Using a serum-free short-term ex vivo expansion system plus optimized rapid and efficient cell immunoadsorption procedures, sufficient primary stage-E1 bone marrow EPC's presently were prepared in sufficient numbers for robust quadruplicate profiling studies.

One related basic point for consideration concerns the choice of stage-E1 CFUe-like cells as a target for EPO-action analyses. Previously, we have shown that with regards to survival effects, EPO can also cytoprotect stage E2 proerythroblasts and stage E3 erythroblasts, but less effectively than for highly-dependent E1 cells [19], [24]. Stage E1 cells also are more stimulated in their proliferative responses by EPO [21], [24]. With regards to profiling per se, several features underscore the likelihood that culled transcripts represent EPO/EPOR response genes. First, among 50 factors assayed by quantitative RT-PCR, all except one (*Pias3*) were confirmed. Second, for the >2-fold modulated set considered, PCA and clustering analyses did not reveal outlying factors. Third, when cluster-4 was independently analyzed, all EPOR targets tested were validated. In addition (through the employ of knocked-in EPOR-H and EPOR-HM alleles) six new EPOR-PY343/Stat5 response genes were independently confirmed including *Tnfr-sf13c*, *Ccrn4l*, *Rpl12*, *Socs2, Chac1* and *Matr3* as novel EPO-regulated factors. Confidence in our presently reported EPO/EPOR- regulated transcriptome is also reinforced via heat-map and p-value distribution analyses.

EPO is best known to promote EPC survival, and expansion [1], [36], and EPO/EPOR-modulated survival therefore is next considered. Among seven such factors, two are novel as *Gas5*, and *Lyl1. Gas5* is of interest in ways that relate to *Pim-1* and *-3.* Specifically *Gas5* can act as a non-coding RNA to regulate mTOR levels [37]. Together, Pim and mTOR pathways reinforce cell survival via pro-metabolic effects [30]. Lyl1, by comparison, is a bHLH transcriptional regulator which recently has been demonstrated to enforce hematopoietic progenitor cell survival [31]. EPO down-modulation of *Bim* (a BH3-only factor) is in-keeping with Bim's role as a Bclx antagonist [38]. Finally, Trib3 and Serpina3g are a pseudokinase, and candidate protease inhibitor each of which recently has been preliminarily demonstrated to affect erythroid progenitor cell survival [19]. For these latter highly EPO-induced factors, LOF studies are in progress (and point to important non-redundant effects exerted in relatively late EPC's) (manuscripts in preparation and submitted).

In maturing CFUe, cell cycle regulation is an atypical affair in that cell division rates accelerate markedly, become synchronous, and then sharply decrease as erythroblasts transition to reticulocytes [39]. In part, this recently has been indicated to involve E2F factor effects [40]. Presently, five EPO-modulated factors are predicted to promote cell cycle progression as Cyclin-D2, Cdc25a and Cyclin b1-interacting protein as induced factors, and Cyclin-G2 plus p27/Kip as repressed inhibitory factors. Btg3, in contrast is a novel EPO-induced factor that previously has been demonstrated to attenuate G2 to M phase transitions [32]. Btg3 therefore may contribute to synchronizing aspects of proerythroblast cell divisions.

In a broad category of additional signal transduction factors, one interesting subset of EPO/EPOR- regulated STF's is negative feedback factors. Among these, suppressors of cytokine signaling Socs-3 previously has been implicated as an EPOR/JAK2 complex inhibitor [34]. In primary bone marrow stage-E1 EPC's, *Socs-3* as well as *Socs-2* are shown to be induced by EPO. *Spreds-1* and *-2,* as also induced by EPO, may well affect not only EPOR complexes, but also RTK's such as Kit and/or EphB4, each of which play erythropoietic roles [41], [42]. Biologically, such suppression might relate, for example, to stage-E1 to -E2 transitions. This brings questions of possible receptor cross-modulation to bear. Eaf1 further is known to suppress Wnt signaling (see Figure 4B).

By extended considerations, select EPO-induced transcriptional events also may result in erythroid cell extrinsic EPC effects. Examples are given by EPO- induction of *Cmtm6* as a CMTM cytokine [43]; *Gdf3* as a candidate inhibitor of TGFbeta [44] and *Oncostatin M* as a newly discovered regulator of hepatic hepcidin and therefore of iron availability [33]. Finally, *Tnfr-sf13C* proved to comprise a strong EPO/EPOR response factor in EPC's and (as discussed below) this has proven to be of clear functional significance.

Tnfr-sf13c to date has been studied in B-cells and (together with its BAFF ligand) is essential for B-cell survival and development [22], [23]. BAFF interestingly is expressed by bone marrow stromal cells, and has been implicated as an anchoring site within marrow for multiple myeloma cells [45]. In an erythropoietic context, we now demonstrate that *Tnfr-sf13c* is a major EPO-response gene in primary bone marrow EPC's, and ligation of Tnfr-sf13c results in not only cytoprotective effects, but also appears to bolster the formation of stage-E3 erythroblasts from stage E1 and E2 progenitors. Our profiling analyses of E1, E2 and E3 EPC's further indicate that these cells are not a significant source of BAFF as compared directly to primary bone marrow stromal cells (data not shown). Therefore, a novel functional connection is implicated between EPC's (via Tnfr-sf13c) and bone marrow stromal cells. It is of potential clinical interest to suggest that BAFF may comprise a rationale anti-anemia agent for possible use in combination with EPO. Via MGI Gene Expression Atlas, twenty-four cited experiments which include *Tnfrsf13c* indicate skewed expression among nine non-hematopoietic tissues (with 7 of 24 studies in brain).

A final set of observations that merit discussion involves additional functional subsets of targets presently discovered to be modulated by EPO. This includes up to 99 EPO modulated genes (see Tables S3, S4, S5, S6, S7, S8). Among these, space (and focus) dictate the brief discussion of only a few such factors. Among transcription factors (Table S3) Eklf3 can both antagonize or substitute for Eklf1, and Kruppel-like factors also can cross-regulate one another [46]. EPO's down-regulation of *Eklf3* therefore might contribute to late erythroid development (ie, by indirectly modulating Eklf1 activation of late erythroid genes, eg, beta- globins). Mllt3, a transcription factor recently shown in Mllt3-null and transgenic mice to regulate early stage erythro- and megakaryopoiesis [47], also was down modulated 3.3 fold by EPO (Table S3). This therefore may favor advancement to late-stage erythroid development. In a category of ubiquitinylation (Table S4), EPO induced the expression of *Usp12* and *Fbxw7*. Usp12 is a deubiquitinating enzyme previously implicated in Fanconi anemia, while Fbxw7 (a substrate binding component of Ub ligase complexes) can act as a tumor suppressor in part by regulating Mcl1 levels [48]. Other EPO- modulated metabolic factors of note (Table S4) include *Tim-9* and *Tim-10* which act coordinately to facilitate transport of hydrophobic proteins to inner mitochondrial membranes [49]. Interestingly, EPO induced mitochondrial biogenesis also has recently been reported in myocardial tissue [15]. In a related category of transporters (Table S4) EPO also regulated the expression of *Slc40a1*, an iron transporter that may correspond to erythroid ferroportin [50]. Here, down- modulation of an iron exporter would result in increased iron accumulation prior to hemoglobinization. Finally, eleven factors involved in ribosome biogenesis were induced by EPO (see Table S6) (for references, also see Table S6). This was unexpected, but of significant interest based on association of mutations in several ribosomal factors with Diamond-Blackfan anemia and 5q-linked myelodysplastic syndrome [51], [52]. In part, this appears to involve a heightened sensitivity of erythroid progenitors to dysregulated ribosome biogenesis (and a consequential activation of p53) [53]. Among EPO/EPOR response factors presently discovered in bone marrow derived CFUe-like progenitors, Rrs1, Rpl12, Urb2 and Bxlc2 are known ribosome biogenic factors; Tsr1, Bysl and Nmd3 are involved in 20S, 40S, and 60S rRNA/subunit formation (respectively); Mak16p and Rbp1B and rRNA stabilizers and/or processing components; Rps24 is a structural component; while Dimt1l is an 18S rRNA adenosine demethylase. To date, none of the above EPO/EPOR targets have been directly implicated in the anemia of MDS or Diamond Blackfan disease. It nonetheless is compelling to suggest that EPO/EPOR signals likely impact on ribosome formation within (pro)erythroblasts – And that this bolstering of ribosome biogenesis may be important to avoid anemia, and keep pace with high demands for the translation of globin transcripts plus erythrocyte membrane and cytoskeletal components.

## Supporting Information

Figure S1 **Transcription factor binding site representation among EPO regulated genes within murine bone marrow- derived CFUe-like progenitors.** For the EPO- modulated genes in K-means clusters #1 – #4 (see Figure 2) DiRE algorithms were applied to identify (and score) enriched transcription factor binding sites. To further inform, results of DiRE analyses are also tabulated on a single gene basis for each EPO- modulated gene within each cluster (see Table S2).
(TIF)

Figure S2 **EPO- modulated survival factors, and cell cycle regulators in CFUe- like EPC's.** Quantitative RT-PCR data for this sub-set of EPO- modulated transcripts are illustrated in bar-graph format.
(TIF)

Figure S3 **EPO- modulated cytokines/receptors, and negative feedback factors in CFUe- like EPC's.** Quantitative RT-PCR data for this subset of EPO- modulated transcripts are illustrated in bar-graph format.
(TIF)

Figure S4 **BAFF inhibition of the apoptosis of primary bone marrow** (**pro**)**erythroblasts.** Primary bone marrow erythroid progenitors were expanded (SP34ex culture). At day-3, EPC's (erythroid progenitor cells) were washed thrice, and returned to culture for 15 hours in the absence of SCF and EPO, but presence of BAFF at moderate [500ng/ml (+)], low [50 ng/ml (++)], or 0 ng/ml [-]. Frequencies of apoptotic EPC's then were determined by Annexin-V staining and flow cytometry. For gated Ter119^pos^ erythroblasts, primary staining profiles are shown. (For CD71^high^ EPC's overall, similar dose-dependent effects on survival also were observed, data not shown).
(TIF)

Table S1 **Quantitative Pcr Primer Pairs.**
(PDF)

Table S2 **Summary Of Epo- Modulated Genes Within K-Means Clusters #1 – #4, Including Candidate Transcription Factor Binding Sites** (**As Predicted Via Dire**)**.**
(PDF)

Table S3 **Epo Modulated Transcription Factors.**
(PDF)

Table S4 **Epo/Epor Modulated Metabolism.**
(PDF)

Table S5 **Epo/Epor Modulated Cancer Biology.**
(PDF)

Table S6 **Epo/Epor Modulated Ribosome Biosynthesis.**
(PDF)

Table S7 **Epo/Epor Modulated Rna Processing.**
(PDF)

Table S8 **Epo/Epor Modulated Other.**
(PDF)

Table S9 **Candidate Regulators Of** ***Tnfrsf13c*** **Expression.**
(PDF)
