# Supplementary figures and images for: Defining an EPOR- Regulated Transcriptome for Primary Progenitors, including Tnfr-sf13c as a Novel Mediator of EPO- Dependent Erythroblast Formation
Source: PLoS One. 2012 Jul 13;7(7):e38530. doi: 10.1371/journal.pone.0038530 (PMC3396641; doi:10.1371/journal.pone.0038530)

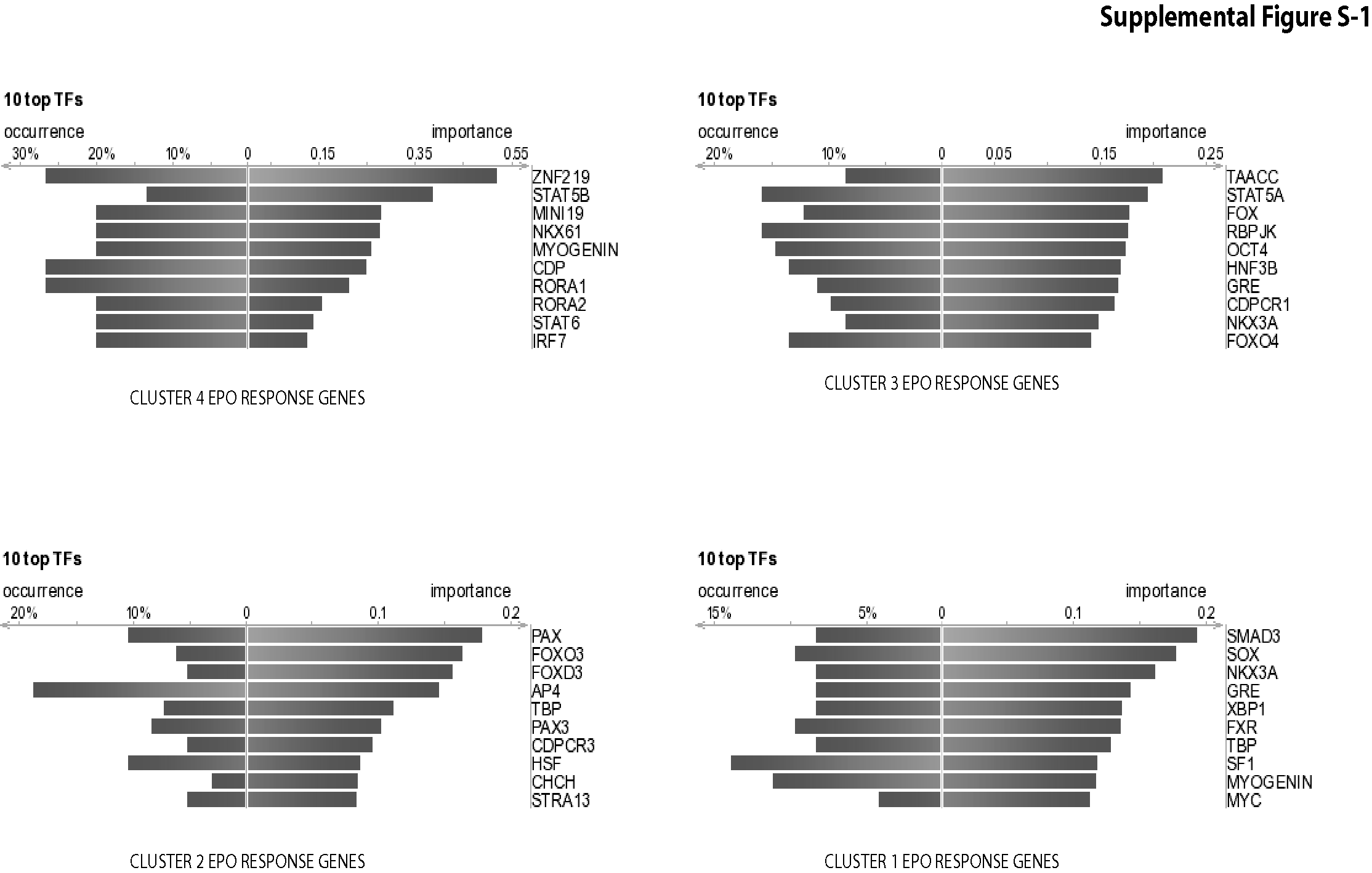

Supplement: Figure S1 — Transcription factor binding site representation among EPO regulated genes within murine bone marrow- derived CFUe-like progenitors. For the EPO- modulated genes in K-means clusters #1 – #4 (see Figure 2) DiRE algorithms were applied to identify (and score) enriched transcription factor binding sites. To further inform, results of DiRE analyses are also tabulated on a single gene basis for each EPO- modulated gene within each cluster (see Table S2). (TIF) [file pone.0038530.s001.tif]

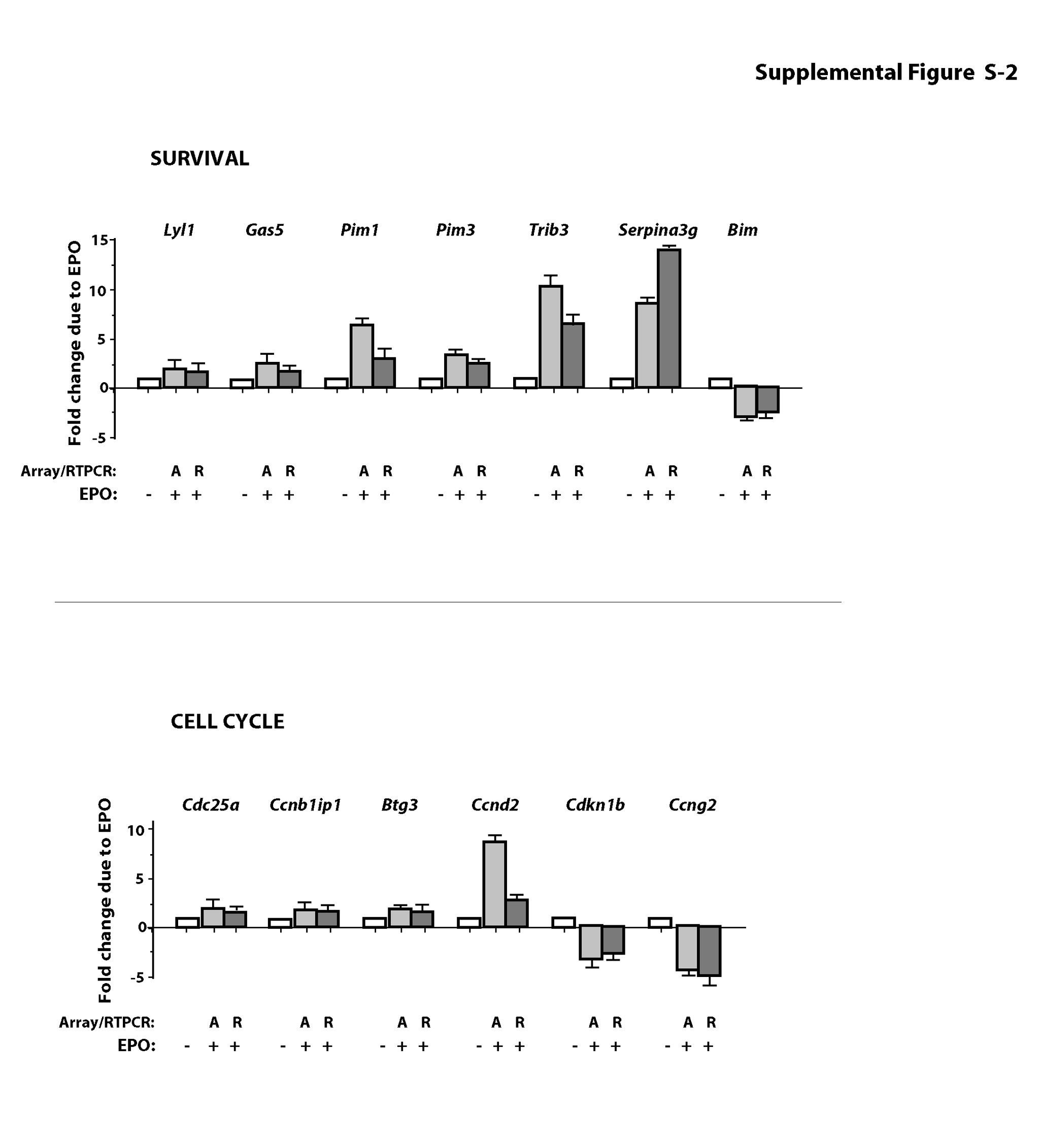

Supplement: Figure S2 — EPO- modulated survival factors, and cell cycle regulators in CFUe- like EPC's. Quantitative RT-PCR data for this sub-set of EPO- modulated transcripts are illustrated in bar-graph format. (TIF) [file pone.0038530.s002.tif]

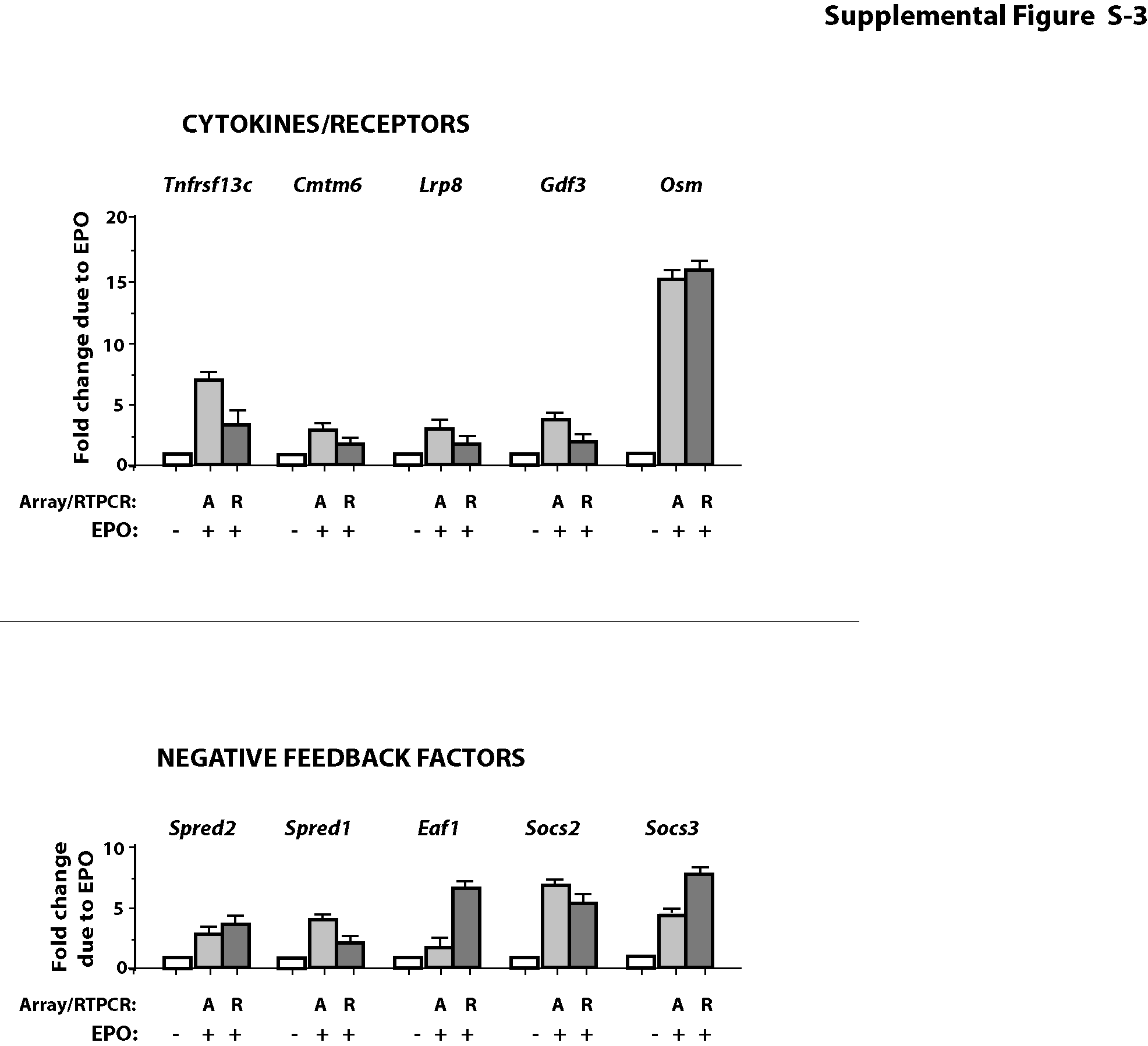

Supplement: Figure S3 — EPO- modulated cytokines/receptors, and negative feedback factors in CFUe- like EPC's. Quantitative RT-PCR data for this subset of EPO- modulated transcripts are illustrated in bar-graph format. (TIF) [file pone.0038530.s003.tif]

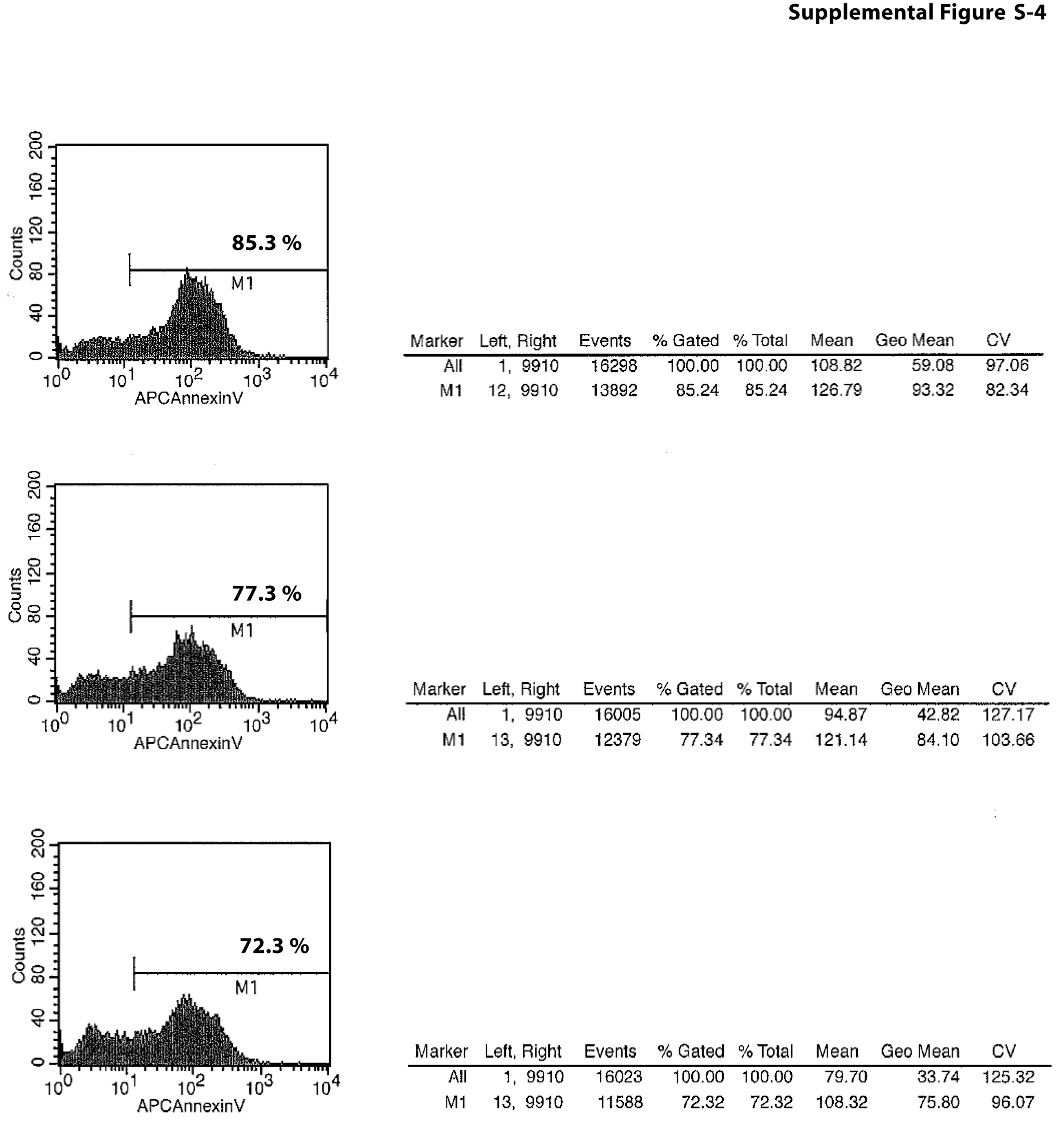

Supplement: Figure S4 — BAFF inhibition of the apoptosis of primary bone marrow (pro)erythroblasts. Primary bone marrow erythroid progenitors were expanded (SP34ex culture). At day-3, EPC's (erythroid progenitor cells) were washed thrice, and returned to culture for 15 hours in the absence of SCF and EPO, but presence of BAFF at moderate [500ng/ml (+)], low [50 ng/ml (++)], or 0 ng/ml [-]. Frequencies of apoptotic EPC's then were determined by Annexin-V staining and flow cytometry. For gated Ter119pos erythroblasts, primary staining profiles are shown. (For CD71high EPC's overall, similar dose-dependent effects on survival also were observed, data not shown). (TIF) [file pone.0038530.s004.tif]
