# Supplementary material for: Defining an EPOR- Regulated Transcriptome for Primary Progenitors, including Tnfr-sf13c as a Novel Mediator of EPO- Dependent Erythroblast Formation
Source: PLoS One. 2012 Jul 13;7(7):e38530. doi: 10.1371/journal.pone.0038530 (PMC3396641; doi:10.1371/journal.pone.0038530)
Supplement: Table S1 — Quantitative Pcr Primer Pairs. (PDF) [file pone.0038530.s005.pdf]

SUPPLEMENTAL TABLE S1: QUANTITATIVE PCR PRIMER PAIRS

Gene Name	Catalog # (SA Bioscience)	Figure #
Matr3	PPM33513A	2D
Chac1	PPM28288A	2D
Ccrn4L	PPM30172A	2D
Socs2	PPM03554A	2D
Tnfrs-13c	PPM34439E	2D
Rpl12	PPM35036A	2D
Lyl1	PPM25208E	3B
Gas5	PPM36248A	3B
Pim1	PPM05401A	3B
Pim3	PPM36664E	3B
Trib3	PPM06271E	3B
Serpina3g	PPM36028A	3B
Bim (Bcl2l11)	PPM03429E	3B
Cdc25a	PPM03246E	3C
Cyclin B1-IP-1	PPM25318A	3C
Btg3	PPM24867A	3C
Ccnd2	PPM02900E	3C
p27/kip1	PPM02909B	3C
Ccng2	PPM03261A	3C
Tnfrs-13c	PPM34439E	4A
Cmtm6	PPM27385A	4A
Lrp8	PPM04129A	4A
Gdf3	PPM04446A	4A
Osm	PPM05385A	4A
Spred2	PPM36075A	4B
Spred1	PPM34660A	4B
Eaf1	PPM28472A	4B
Socs2	PPM03554A	4B
Socs3	PPM05161A	4B
