# Supplementary material for: Defining an EPOR- Regulated Transcriptome for Primary Progenitors, including Tnfr-sf13c as a Novel Mediator of EPO- Dependent Erythroblast Formation
Source: PLoS One. 2012 Jul 13;7(7):e38530. doi: 10.1371/journal.pone.0038530 (PMC3396641; doi:10.1371/journal.pone.0038530)
Supplement: Table S2 — Summary Of Epo- Modulated Genes Within K-Means Clusters #1 – #4, Including Candidate Transcription Factor Binding Sites (As Predicted Via Dire). (PDF) [file pone.0038530.s006.pdf]

## SUPPLEMENTAL TABLE S2: SUMMARY OF EPO- MODULATED GENES WITHIN K-MEANS CLUSTERS #1 - #4, INCLUDING CANDIDATE TRANSCRIPTION FACTOR BINDING SITES (as predicted via DiRE).

## CLUSTER 4 EPO RESPONSE GENES

| # | Regulatory element | Type | Score | Locus | Gene | Candidate transcription factor binding sites (relative positions) |
|---|---|---|---|---|---|---|
| 1 | chr10:94880055-94880493 | promoter | 0.406 | chr10:94786738-94943373 | Socs2 | 6 :: WT1(96) ZF5(216) AREB6(224) RORA2(415) RORA1(431) RORA2(431) |
| 2 | chr11:4136272-4136885 | UTR5 | 0.000 | chr11:4122426-4157560 | Osm | 42 :: R(61) NF1(81) R(90) AML(97) TAL1ALPHAE47(112) TAL1BETAE47(112) PAX6(155) SMAD4(168) SREBP(303) AML(325) VBP(332) CREBATF (333) ROAZ(340) EGR1(341) NGFIC(341) TAXCREB(354) PAX6(358) RORA1(358) IRF7(372) STAT5B(374) ZF5(393) ZF5(414) NANOG(418) ACAAT (422) CDP(443) RORA1(449) SREBP(452) P53_DECAMER(454) UF1H3BETA(477) ZNF219(479) MZF1(481) UF1H3BETA(483) WT1(483) ZNF219 (486) MZF1(487) UF1H3BETA(488) CACD(492) SPZ1(495) HOXA7(517) SPZ1(520) SMAD4(542) TAXCREB(575) |
| 3 | chr11:117831309-117831444 | promoter | 0.225 | chr11:117734826-117848170 | Socs3 | 1 :: ZF5(90) |
| 4 | chr12:105415954-105416340 | intergenic | 0.126 | chr12:105392117-105480144 | Serpina3f, Serpina3g | 3 :: LMO2COM(114) GCNF(153) STAT6(300) |
| 5 | chr15:61815830-61816035 | promoter | 1.072 | chr15:60817091-62821892 | Myc | 3 :: STAT6(4) UF1H3BETA(22) TAXCREB(129) |
| 6 | chr18:35729880-35731677 | UTR5 | 0.000 | chr18:35658523-35758318 | Matr3 | 36 :: CRX(92) P53_DECAMER(131) CDP(338) ACAAT(347) CDPCR1(356) AREB6(856) AIRE(862) STAT6(863) CIZ(879) OCT4(1051) XFD1(1127) GFI1(1136) NKX61(1143) MRF2(1189) HNF4_DR1(1207) OCT4(1213) CMAF(1217) PAX6(1221) RORA1(1221) RORA2(1221) GFI1(1222) GCNF (1222) ZEC(1351) NFKAPPAB65(1396) NFKAPPAB(1396) VMAF(1452) AFP1(1487) CEBPGAMMA(1528) MMEF2(1608) DR3(1662) CDPCR1(1664) CDP(1665) AIRE(1669) RORA2(1707) CDP(1768) MMEF2(1774) |
| 7 | chr2:32816491-32816655 | promoter | 0.001 | chr2:32815708-32828498 | Rpl12 | 2 :: CACD(35) NFKAPPAB50(91) |
| 8 | chr2:119176884-119177138 | UTR5 | 0.001 | chr2:119161397-119198772 | Chac1 | 22 :: FXR(34) E2F1DP2(54) MZF1(58) CACD(58) UF1H3BETA(60) MYOGENIN(76) E47(77) LMO2COM(77) AP4(77) TAL1(77) ZF5(123) MINI19(138) MINI20(138) AP4(143) PAX6(145) PAX6(164) EBOX(170) MYOGENIN(170) AREB6(171) LMO2COM(171) E47(172) ZF5(197) |
| 9 | chr2:152163094-152163464 | UTR3 | 0.857 | chr2:152158388-152192540 | Trib3 | 2 :: R(237) NF1(344) |
| 10 | chr3:50394274-50395813 | intergenic | 0.000 | chr3:50247543-51059575 | Ccrn4l | 29 :: XPF1(97) NKX61(135) IRF7(152) ISRE(153) NFAT(157) FOXJ2(226) CDP(319) TAL1ALPHAE47(369) TAL1BETAE47(369) XPF1(474) LEF1TCF1(549) TBP(938) AREB6(987) HAND1E47(1012) GZF1(1068) MYOGENIN(1113) ALX4(1168) RSRFC4(1190) CHOP(1207) OCT4(1261) FOXJ2(1295) T3R(1352) IK1(1357) RORA2(1373) HOXA4(1375) NKX62(1376) FXR(1403) LHX3(1481) AFP1(1483) |
| 11 | chr6:31731002-31731975 | intergenic | 0.000 | chr6:31459491-32000233 | Podxl | 25 :: PAX6(15) FOXJ2(184) IRF7(190) STAT4(194) STAT5B(198) CDP(201) LMO2COM(207) MZF1(231) ACAAT(292) T3R(418) LHX3(425) OTX(426) CRX(428) GFI1(438) NKX61(444) HOXA4(503) RORA1(540) CEBPGAMMA(565) LEF1TCF1(694) SREBP(864) TAXCREB(865) ATF3(867) COUP_DR1(870) HNF4_DR1(870) PPAR_DR1(870) |
| 12 | chr6:127101196-127101402 | promoter | 0.164 | chr6:127059565-127403705 | Ccnd2 | 12 :: MYOGENIN(19) LMO2COM(20) AP4(20) TAL1(20) E47(21) ZNF219(56) CACD(58) ZNF219(63) MINI19(112) MINI20(112) AML(140) PEBP(140) |
| 13 | chr7:133768949-133769198 | UTR5 | 0.001 | chr7:133738432-133792873 | Nupr1 | 5 :: CHOP(144) ZNF219(214) MINI19(232) MINI20(232) AP4(243) |
| 14 | chr8:11008431-11008487 | promoter | 1.227 | chr8:10634046-11198380 | Irs2 | 1 :: ZNF219(56) |
| 15 | chr9:107198066-107198188 | promoter | 0.393 | chr9:107192226-107229941 | Cish | 2 :: HAND1E47(26) XPF1(70) |

# CLUSTER 3 EPO RESPONSE GENES

| # | Regulatory element | Type | Score | Locus | Gene | Candidate transcription factor binding sites (relative positions) |
|---|---|---|---|---|---|---|
| 1 | chr1:46863894-46866797 | intergenic | 0.260 | chr1:45883712-46864387 | Slc40a1 | 11 :: RORA2(7) AP1(85) TBX5(91) ZEC(159) CEBPB(165) ACAAT(208) XPF1(255) ZIC1(258) BRCA(260) CREBATF(329) NKX25(416) |
| 2 | chr1:58477056-58477926 | intron | 0.001 | chr1:58463409-58483425 | Clk1 | 28 :: CREBP1(7) USF(12) NKX25(12) KROX(14) NGFIC(14) SMAD4(41) COMP1(42) ACAAT(44) ALPHACP1(45) CDPCR1(47) DEC(60) ATF1 (66) IRF7(79) CEBPDELTA(79) MZF1(94) TAACC(116) ZBRK1(138) CAP(154) STAT3(159) STAT5A(159) STAT5A(216) TAL1BETAE47(216) GATA(226) BRCA(251) FOXO4(262) HNF3B(363) XFD1(363) XFD3(434) |
| 3 | chr1:141349747-141349938 | promoter | 0.233 | chr1:141349785-141398242 | Aspm | 2 :: MMEF2(102) MZF1(174) |
| 4 | chr1:174124804-174125729 | UTR3 | 1.008 | chr1:174065935-174126804 | Wdr42a | 29 :: TBX5(7) BRACH(11) SMAD4(31) PAX4(283) CRX(304) GATA4(312) EGR(325) PAX4(328) MZF1(329) MOVOB(329) EGR(331) ALPHACP1(375) TAACC(388) IRF1(389) RORA1(392) SOX9_B1(406) STAT5A(474) NFKAPPAB(486) PEBP(557) EGR(652) MZF1(653) ACAAT(714) GATA(733) CDP(764) RSRFC4(766) FREAC7(766) TBP(766) NKX3A(767) NKX61(773) |
| 5 | chr10:41600939-41601649 | intergenic | 0.780 | chr10:41536197-41634422 | Sesn1 | 9 :: ATF1(404) NKX25(404) ZEC(445) GRE(454) SMAD(502) AP1(523) ATF4(524) CREBP1(524) NRF2(525) |
| 6 | chr10:41605964-41606159 | promoter | 0.812 | chr10:41536197-41634422 | Sesn1 | 2 :: CAP(160) IRF1(162) |
| 7 | chr10:57886990-57887512 | UTR3 | 0.379 | chr10:57770634-57909596 | Lims1 | 8 :: CEBPGAMMA(120) POU1F1(125) SOX9_B1(131) RORA1(253) RORA2(253) AIRE(283) CDP(377) XFD2(430) |
| 8 | chr10:74980056-74980200 | UTR5 | 0.010 | chr10:74903767-74981083 | 1110038D17Rik | 6 :: KROX(2) ETF(6) MINI20(14) ETF(51) MAZ(97) ETF(98) |
| 9 | chr11:22797527-22798111 | intergenic | 0.863 | chr11:22759724-22890592 | Commd1 | 5 :: XFD3(84) RORA1(225) GZF1(250) SEF1(342) RFX1(487) |
| 10 | chr11:22882537-22882825 | promoter | 1.719 | chr11:22759724-22890592 | Commd1 | 2 :: NFMUE1(38) NKX61(65) |
| 11 | chr11:94188005-94188644 | intergenic | 1.790 | chr11:94103925-94189305 | 3300001P08Rik | 6 :: TAACC(136) NKX25(179) GR(219) GRE(219) RBPJK(229) FOX(250) |
| 12 | chr11:98768251-98768497 | promoter | 2.475 | chr11:98764968-98798998 | Cdc6 | 3 :: RORA1(12) CDX2(164) PBX(222) |
| 13 | chr12:15823752-15823839 | promoter | 0.847 | chr12:14799513-16543433 | Trib2 | 3 :: PBX(16) CDPCR1(17) ACAAT(18) |
| 14 | chr12:15824292-15824420 | promoter | 1.196 | chr12:14799513-16543433 | Trib2 | 2 :: EGR(78) MTF1(79) |
| 15 | chr12:15824737-15824839 | promoter | 1.825 | chr12:14799513-16543433 | Trib2 | 2 :: NKX3A(49) NKX3A(55) |
| 16 | chr12:25644026-25644715 | UTR3 | 0.002 | chr12:25401263-25657431 | Mboat2 | 14 :: CREL(123) NFKAPPAB(123) HOXA7(140) MAZ(164) EGR1(167) NGFIC(167) RFX(270) OTX(316) OCT4(348) DBP(447) CHOP(513) CDX2(516) FOXO4(519) FREAC2(582) |
| 17 | chr12:32636334-32636645 | promoter | 0.762 | chr12:32398747-32643179 | Hbp1 | 2 :: OCT4(49) STAT5A(233) |
| 18 | chr12:112681946-112682104 | promoter | 0.011 | chr12:112669755-112722268 | Tnfaip2 | 2 :: EGR(15) RBPJK(29) |
| 19 | chr13:94859070-94859968 | intron | 1.027 | chr13:94717223-94971387 | Lhfpl2 | 5 :: GRE(111) COUP_DR1(248) STAT3(359) PAX4(480) XVENT1(554) |
| 20 | chr13:104555591-104556888 | intergenic | 0.011 | chr13:104554663-104813353 | Erbb2ip | 13 :: XPF1(68) GR(72) CEBPB(80) SOX9_B1(131) GZF1(170) TBP(183) GEN_INI2(685) BRCA(767) HMGIY(775) BRCA(850) NKX3A(923) GRE(1090) IRF1(1288) |
| 21 | chr13:120273301-120273637 | intron | 0.006 | chr13:120249162-120274924 | 4833420G17Rik | 6 :: CMAF(103) AP1(115) TBP(157) PXR(200) NKX3A(236) CRX(329) |
| | | | | | | 51 :: PAX4(156) ACAAT(364) PEBP(366) NKX61(453) OCT4(458) HMGIY(482) HNF6(509) RSRFC4(514) TBX5(558) PADS(594) SEF1(602) |

| | | | | | | |
|---|---|---|---|---|---|---|
| 22 | chr14:76838223-76840184 | intron | 0.008 | chr14:76539983-77423955 | Tsc22d1 | ATF1(610) ATF4(611) CREBP1(611) CREBATF(611) CREBP1CJUN(611) RBPJK(674) E2F1DP1(675) FREAC2(687) XFD2(688) FOXO4 (690) CREL(700) XFD1(710) FOX(711) DEC(714) E2A(792) HMGIY(868) TBP(1076) PLZF(1205) STAT5A(1315) RFX1(1374) ACAAT(1470) ATF4(1487) AP1(1487) STAT5A(1498) XVENT1(1520) GR(1523) STAT4(1542) E12(1630) E2A(1633) AP1(1693) USF(1702) E2A(1705) IRF2 (1762) OTX(1783) COMP1(1810) CDX2(1816) XFD1(1819) CAP(1838) CETS168(1850) ACAAT(1909) |
| 23 | chr15:36260740-36261583 | intergenic | 0.010 | chr15:36168752-36407383 | Rnf19a | 6 :: OTX(359) MMEF2(361) NKX61(363) POU1F1(499) HOX13(552) XVENT1(552) |
| 24 | chr16:20124823-20125817 | UTR3 | 2.554 | chr16:19983067-20137083 | Klhl24 | 9 :: CDP(21) IRF7(108) CDP(160) STAT5A(373) CEBPDELTA(577) RBPJK(600) BRCA(740) TBP(981) GATA(982) |
| 25 | chr16:22811429-22811924 | intergenic | 1.790 | chr16:22811495-22858708 | Tbccd1 | 12 :: NRF2(108) VMAF(109) AP1(109) AP1(133) RORA2(134) CDPCR1(157) PBX(158) PITX2(182) CRX(183) VMAF(224) NRF2(254) AP1 (255) |
| 26 | chr16:23989664-23989957 | promoter | 0.646 | chr16:23930905-24393575 | Bcl6 | 6 :: MOVOB(7) IRF7(40) AIRE(107) XFD3(159) HMGIY(172) NGFIC(195) |
| 27 | chr17:28936470-28936677 | promoter | 2.831 | chr17:28918967-28995311 | Brpf3 | 6 :: HNF3B(63) NFKAPPAB(80) PBX(93) OTX(95) TBX5(106) CEBPB(183) |
| 28 | chr17:28937250-28937366 | promoter | 3.192 | chr17:28918967-28995311 | Brpf3 | 6 :: RBPJK(54) EFC(82) SMAD(97) HP1SITEFACTOR(105) RUSH1A(109) XFD3(116) |
| 29 | chr18:38530135-38530833 | intergenic | 0.012 | chr18:38477524-38578597 | Gnpda1 | 27 :: HMGIY(40) IRF7(42) GATA(93) XPF1(95) FOXO4(147) FREAC2(148) FREAC7(148) OCT4(159) IRF7(178) STAT5A(216) STAT3(224) STAT5A(224) CETS168(226) NERF(228) NKX25(233) EFC(270) PAX4(277) POU1F1(279) CRX(286) HNF6(296) PXR(301) AIRE(304) RORA2(306) FREAC2(357) FOXO4(358) FOX(358) HNF3B(359) |
| 30 | chr18:70083849-70084061 | promoter | 0.001 | chr18:69847676-70138698 | Ccdc68 | 4 :: IRF7(22) IRF1(23) IRF2(23) HMGIY(71) |
| 31 | chr19:3385436-3385777 | UTR3 | 0.012 | chr19:3296161-3386882 | Cpt1a | 4 :: HMGIY(58) XFD1(208) STAT5A(240) PITX2(286) |
| 32 | chr19:30103569-30104000 | promoter | 0.359 | chr19:30080513-30172895 | Uhrf2 | 3 :: OCT4(16) HMGIY(335) PADS(357) |
| 33 | chr19:30167355-30168332 | UTR3 | 0.824 | chr19:30080513-30172895 | Uhrf2 | 19 :: MAZ(62) MZF1(64) E2F1DP1(119) RBPJK(120) RFX(162) PLZF(264) FOX(264) CEBPB(270) GR(285) PLZF(341) CEBPB(498) SOX9_B1 (593) RFX1(614) MYCMAX(626) PADS(630) CEBPB(783) CEBPDELTA(783) HNF3B(843) CDX2(848) |
| 34 | chr19:53674797-53674946 | UTR5 | 0.004 | chr19:53615844-53966718 | Smc3 | 7 :: ETF(75) EGR(76) KROX(77) EGR1(77) NGFIC(77) NFMUE1(91) KROX(146) |
| 35 | chr2:73119315-73120504 | intergenic | 0.910 | chr2:73113401-73152136 | 1700023B02Rik | 10 :: ZBRK1(410) CEBPB(496) AP1(605) PAX4(609) FREAC2(632) FOXO4(633) MMEF2(673) NKX25(846) ZIC3(1094) HMGIY(1147) |
| 36 | chr2:119449600-119449853 | intron | 2.521 | chr2:119442827-119478733 | Nusap1 | 4 :: COMP1(10) E2F1DP1(97) BRACH(103) TBX5(107) |
| 37 | chr2:127950515-127950699 | promoter | 0.954 | chr2:127949700-128438427 | Bcl2l11 | 3 :: NERF(47) NFKAPPAB(119) KROX(172) |
| 38 | chr2:130665938-130666012 | promoter | 0.008 | chr2:130523286-130732224 | 4930402H24Rik | 1 :: MTF1(10) |
| 39 | chr3:68650928-68651812 | intergenic | 0.010 | chr3:68502448-68809602 | Ift80 | 21 :: PAX4(113) CRX(120) MYCMAX(171) XPF1(182) ZTA(257) HP1SITEFACTOR(323) RORA1(449) RORA2(449) AP1(473) POU1F1(558) SMAD(614) CHOP(638) ZBRK1(640) PXR(640) ZIC1(645) IRF1(696) HNF6(805) RORA1(809) RORA2(809) NKX61(867) OCT4(873) |
| 40 | chr3:95391276-95391605 | promoter | 0.662 | chr3:95391530-95428893 | Golph3l | 2 :: GATA(263) HNF6(306) |
| 41 | chr3:95391634-95391824 | promoter | 1.817 | chr3:95391530-95428893 | Golph3l | 4 :: HNF3B(52) FREAC2(53) PLZF(67) PLZF(101) |
| 42 | chr3:151846974-151847753 | UTR3 | 0.280 | chr3:151828865-151874289 | Dnajb4 | 12 :: TBX5(115) PAX4(118) STAT5A(123) XFD1(156) TBP(157) TBX5(224) HMGIY(255) E4BP4(260) CREBP1(260) PITX2(313) CREL(483) NFKAPPAB(484) |

| | | | | | | |
|---|---|---|---|---|---|---|
| 43 | chr3:151873358-151873556 | promoter | 1.706 | chr3:151828865-151874289 | Dnajb4 | 6 :: ETF(65) CREBP1(73) NFMUE1(82) CEBPB(87) NFMUE1(112) NFMUE1(118) |
| 44 | chr3:151873665-151873739 | promoter | 0.692 | chr3:151828865-151874289 | Dnajb4 | 3 :: MYCMAX(18) ETF(22) STAT3(27) |
| 45 | chr4:11130590-11131613 | UTR3 | 0.007 | chr4:11101541-11131926--chr4:11126305-11192145 | Ccne2--Ints8 | 22 :: XVENT1(153) PLZF(169) CEBPB(198) E4BP4(198) CEBPGAMMA(200) AP1(211) TBP(216) GRE(345) FREAC2(379) FREAC7(379) FOXO4(380) XFD2(380) NFKAPPAB(418) RFX1(441) ZTA(603) RORA1(630) RORA2(630) ATF1(827) FOXO4(873) PADS(921) FREAC2(970) FOX(970) |
| 46 | chr4:11152951-11153319 | intron | 0.362 | chr4:11126305-11192145 | Ints8 | 2 :: HNF3B(125) FOX(126) |
| 47 | chr4:11188595-11189170 | intergenic | 2.587 | chr4:11126305-11192145 | Ints8 | 3 :: CMAF(315) GRE(322) OCT4(346) |
| 48 | chr4:24432154-24432619 | UTR5 | 0.005 | chr4:23432311-24544401 | F730047E07Rik | 3 :: CREBP1CJUN(326) ALPHACP1(386) PAX4(424) |
| 49 | chr4:33377072-33377572 | intergenic | 0.126 | chr4:33321479-33397283 | Pnrc1 | 7 :: IRF1(59) STAT5A(96) STAT5A(104) MINI20(178) RFX1(197) ZTA(234) STAT5A(362) |
| 50 | chr4:94357310-94358275 | intergenic | 2.551 | chr4:94269948-94403090 | Lrrc19 | 19 :: PXR(117) CMAF(145) AP1(147) SOX9_B1(284) STAT3(386) STAT5A(386) IRF1(422) XPF1(498) ATF1(533) XPF1(537) CETS168(539) ZEC(548) PBX(548) CDPCR1(549) CEBPDELTA(584) GRE(590) MTF1(635) SMAD4(718) NKX3A(885) |
| 51 | chr4:94363469-94363913 | intergenic | 0.093 | chr4:94269948-94403090 | Lrrc19 | 9 :: SOX9_B1(13) NFKAPPAB(153) HMGIY(153) RBPJK(385) GATA(412) FREAC7(427) FOX(428) XFD2(428) XFD3(428) |
| 52 | chr4:134482963-134483294 | UTR3 | 3.407 | chr4:134470864-134486894 | D4Wsu53e | 6 :: OCT4(155) GR(172) NFMUE1(176) TBX5(198) HMGIY(256) FOX(294) |
| 53 | chr5:8059513-8060113 | intron | 0.134 | chr5:7984228-8072097 | Sri | 5 :: ZIC1(149) ZIC3(149) ZIC3(154) ZEC(174) CEBPDELTA(181) |
| 54 | chr5:64813890-64814897 | intergenic | 0.008 | chr5:64742754-65316032 | Klf3 | 23 :: RORA1(83) IRF1(120) COMP1(219) STAT3(267) IRF7(270) RFX(291) RFX1(295) CRX(401) E2F1DP1RB(454) ZEC(462) NKX25(463) CDPCR1(482) CRX(506) GR(553) RBPJK(590) FOXO4(636) USF(657) MYCMAX(657) MYCMAX(664) USF(664) COUP_DR1(665) XFD1(714) NERF(829) |
| 55 | chr5:93650595-93651290 | intergenic | 0.009 | chr5:93635055-93947516 | Ccng2 | 8 :: XFD1(86) FREAC7(87) HNF6(88) CDX2(100) NERF(480) CETS168(482) STAT3(485) ZEC(545) |
| 56 | chr5:110732374-110732593 | intron | 0.006 | chr5:110714919-110768651 | Pole | 3 :: GR(94) SMAD4(113) SZF11(158) |
| 57 | chr5:139725497-139725882 | UTR3 | 0.004 | chr5:139663800-139728323 | Unc84a | 12 :: HMGIY(57) PAX4(91) PAX4(100) HNF3B(107) HNF6(109) MMEF2(113) GATA(128) CEBPB(138) HP1SITEFACTOR(244) GZF1(248) MAZ(329) DBP(348) |
| 58 | chr5:145952385-145952496 | intergenic | 1.191 | chr5:145952464-145966109 | Zkscan14 | 2 :: DEC(94) RORA2(101) |
| 59 | chr5:148706410-148706512 | promoter | 0.507 | chr5:148689557-148768892 | Slc46a3 | 3 :: CDPCR1(69) HOXA7(71) MTF1(100) |
| 60 | chr6:39350505-39351208 | intron | 0.953 | chr6:39341105-39412283 | Mkrn1 | 19 :: E2F1DP1(3) BRACH(65) FOXO4(208) TAACC(219) RBPJK(241) SMAD(248) TAACC(263) XBP1(303) NKX25(379) CDP(455) GEN_INI2(513) OCT4(515) ZIC1(536) ZEC(540) STAT4(567) IRF1(669) IRF2(669) SMAD(696) NKX25(702) |
| 61 | chr6:71442383-71442656 | promoter | 1.639 | chr6:71390484-71493846 | Rnf103 | 3 :: RORA1(166) PAX4(191) BRACH(198) |
| 62 | chr6:71445013-71445942 | intron | 2.422 | chr6:71390484-71493846 | Rnf103 | 4 :: ZTA(459) NKX61(481) ACAAT(552) TAACC(673) |

| | | | | | | |
|---|---|---|---|---|---|---|
| 63 | chr6:72022557-72024200 | intergenic | 0.008 | chr6:71929473-72156142 | St3gal5 | 26 :: OCT4(20) RORA1(84) RORA2(84) HNF6(87) GR(137) SOX9_B1(143) POU1F1(335) HP1SITEFACTOR(358) MMEF2(360) NKX61(376) ZTA(632) XPF1(706) TAL1BETAE47(921) HOXA7(929) NFMUE1(1050) NRF2(1159) NKX25(1174) NFMUE1(1233) AP1(1287) RORA2(1290) ACAAT(1387) CDPCR1(1390) RFX1(1448) E2F1DP1RB(1546) E2F1DP1(1546) NRF2(1574) |
| 64 | chr6:87763135-87763397 | promoter | 0.001 | chr6:87730134-87768352 | Rab43 | 2 :: P53(7) RBPJK(134) |
| 65 | chr6:88581637-88582342 | intergenic | 0.002 | chr6:88577443-88785880 | Mgll | 4 :: SMAD(198) CHOP(289) GZF1(433) IRF1(490) |
| 66 | chr6:120307241-120307566 | UTR5 | 0.133 | chr6:120244603-120413233 | Ccdc77 | 2 :: NFMUE1(202) STAT3(285) |
| 67 | chr6:134869745-134869871 | promoter | 0.003 | chr6:134837777-134932012 | Cdkn1b | 2 :: GATA(3) KROX(125) |
| 68 | chr7:28261756-28262268 | UTR3 | 0.089 | chr7:28253153-28271958 | Sertad3 | 10 :: EGR1(146) SMAD(154) TBP(198) SMAD(238) IRF2(258) STAT5A(262) RBPJK(280) PAX4(339) MYCMAX(426) CHOP(431) |
| 69 | chr7:107363803-107364889 | intergenic | 1.015 | chr7:107333454-107434023 | Pgm2l1 | 17 :: RBPJK(51) HNF3B(107) TAL1BETAE47(163) SMAD(190) E4BP4(203) KROX(243) MAZ(246) KROX(249) TAL1BETAE47(263) USF(263) HNF3B(295) CMAF(339) HNF3B(387) SOX9_B1(388) CEBPGAMMA(595) AP1(964) NRF2(965) |
| 70 | chr7:107374745-107375015 | promoter | 0.319 | chr7:107333454-107434023 | Pgm2l1 | 2 :: CHOP(13) GATA4(144) |
| 71 | chr7:117914959-117915555 | promoter | 0.002 | chr7:117773416-117959856 | Ampd3 | 4 :: TBX5(238) MYCMAX(324) APOLYA(339) SMAD(491) |
| 72 | chr7:117957033-117957457 | intergenic | 0.525 | chr7:117955939-117993505 | Rnf141 | 6 :: DBP(144) XFD2(199) HNF3B(200) NKX3A(206) IRF7(220) RFX1(405) |
| 73 | chr7:128108328-128108990 | intron | 0.002 | chr7:128089798-128203862 | Cdr2 | 10 :: RBPJK(130) PEBP(245) EFC(271) GR(344) FOXO4(425) MYCMAX(434) RBPJK(434) OTX(472) CDP(513) CMAF(537) |
| 74 | chr7:138527079-138527352 | intron | 0.001 | chr7:138506271-138532136 | Pstk | 2 :: COUP_DR1(24) PPAR_DR1(24) |
| 75 | chr8:54953810-54954867 | intergenic | 4.472 | chr8:54608795-55162881 | Neil3 | 12 :: PLZF(80) BRCA(398) OTX(553) PITX2(564) GZF1(565) CDPCR1(666) PBX(667) IRF1(690) TBX5(703) GR(763) HNF3B(772) TAACC (1027) |
| 76 | chr9:14019472-14020624 | intergenic | 0.006 | chr9:13651101-14157547 | Sesn3 | 22 :: EFC(373) ZIC1(379) ZIC3(379) BRCA(380) XVENT1(385) VMAF(400) NRF2(401) ZTA(456) VMAF(459) NRF2(460) CEBPDELTA(530) CHOP(570) CEBPB(572) CREBATF(573) SEF1(626) COUP_DR1(638) PPAR_DR1(638) STAT3(729) CEBPDELTA(730) GR(910) XPF1(914) OCT4(942) |
| 77 | chr9:43048862-43048973 | promoter | 0.006 | chr9:43014909-43118840 | Oaf | 2 :: USF(33) CAP(83) |
| 78 | chr9:82759675-82761073 | UTR3 | 0.004 | chr9:82743563-83003506 | Phip | 31 :: HNF6(62) PXR(116) PITX2(136) E4BP4(224) CREBP1(224) FOXO4(362) HNF3B(373) FREAC7(374) FOX(374) CEBPGAMMA(379) TBP (445) RUSH1A(448) STAT5A(457) NKX25(462) POU1F1(464) FOXO4(599) SEF1(658) TAACC(672) AP1(717) RORA2(766) TAACC(849) BRCA(911) RUSH1A(1051) HMGIY(1059) GATA(1073) OCT4(1082) NKX3A(1089) RSRFC4(1128) TBP(1128) AP1(1207) PLZF(1364) |
| 79 | chr9:107661385-107662106 | intron | 0.001 | chr9:107612816-107675887 | Rbm5 | 9 :: NKX25(81) SOX9_B1(244) GR(360) GRE(360) GR(520) XBP1(678) P53(678) CEBPB(716) CREBP1(716) |
| 80 | chr9:120025990-120026183 | intron | 1.463 | chr9:120007378-120036900 | Slc25a38 | 5 :: NERF(40) CETS168(42) STAT5A(92) STAT3(100) STAT5A(107) |
| 81 | chrX:35884632-35885467 | UTR3 | 0.001 | chrX:35809633-35953832 | Cul4b | 13 :: MMEF2(39) RUSH1A(175) AIRE(179) CDX2(180) XVENT1(193) HP1SITEFACTOR(232) ZEC(274) NKX25(363) CEBPB(474) STAT4(585) COUP_DR1(733) USF(733) ZBRK1(734) |

# CLUSTER 2 EPO RESPONSE GENES

| # | Regulatory element | Type | Score | Locus | Gene | Candidate transcription factor binding sites (relative positions) |
|---|---|---|---|---|---|---|
| 1 | chr1:46910506-46910613 | promoter | 0.967 | chr1:45988084-47910238 | Slc39a10 | 3 :: NRF1(41) HIF1(48) IRF1(102) |
| 2 | chr1:93181933-93182790 | UTR3 | 0.302 | chr1:93123943-93193919 | Ube2f | 11 :: PAX3(86) R(212) IPF1(289) NRF1(351) LYF1(480) STAF(485) OCT1(514) FOXD3(588) CIZ(590) PXR(648) AP4(739) |
| 3 | chr1:123939632-123940464 | intergenic | 0.002 | chr1:123425104-124464059 | Ddx18 | 12 :: AP4(216) MYB(256) AHRARNT(258) E47(268) MYB(268) MEF3(339) TST1(382) PU1(411) E4F1(420) HSF2(573) HSF(576) OCT1(633) |
| 4 | chr1:194957752-194958232 | promoter | 0.564 | chr1:194861895-194979305 | AA408296 | 10 :: OCT1(23) LMO2COM(48) AP4(49) MYOGENIN(49) FOXO3(120) FOXO4(120) TTF1(229) DEC(307) TTF1(308) NKX25(308) |
| 5 | chr10:7435096-7436289 | UTR3 | 0.301 | chr10:7398728-7445813 | ##Lats1, NM_010690 | 19 :: TBX5(102) TBP(461) NKX25(481) CREBATF(519) PAX3(587) FOXM1(611) MEIS1BHOXA9(657) FOXD3(674) AP2REP(716) TAACC(749) PLZF(828) ATATA(930) RUSH1A(932) RFX1(958) PLZF(1011) CDX(1082) HNF3ALPHA(1089) CEBP(1096) NKX25(1163) |
| 6 | chr10:39862357-39862885 | intergenic | 0.912 | chr10:39862069-39966845 | Bxdc1 | 2 :: CDX2(142) OCT1(423) |
| 7 | chr10:40022470-40022902 | promoter | 0.001 | chr10:39977472-40069142 | Amd2 | 9 :: PAX3(44) HIF1(52) AHRHIF(53) CEBPGAMMA(169) USF(193) HIF1(194) ZEC(203) SPZ1(231) MYB(310) |
| 8 | chr10:61110627-61110752 | promoter | 0.008 | chr10:61045121-61143068 | Ppa1 | 1 :: HSF(90) |
| 9 | chr10:77178475-77178964 | intergenic | 1.856 | chr10:77081518-77183316 | Ube2g2 | 19 :: USF(78) E47(90) TAL1ALPHAE47(90) AP4(90) TAL1(90) LBP1(90) MYOGENIN(91) HIF1(152) TAL1(159) AP4(160) KAISO(164) KAISO(176) TAL1(189) PAX(268) AHRARNT(283) KAISO(299) KAISO(356) SPZ1(381) NERF(429) |
| 10 | chr10:121024312-121025039 | UTR3 | 0.011 | chr10:121023805-121078703 | Xpot | 10 :: POLY(136) IPF1(223) XBP1(229) TFE(310) COREBINDINGFACTOR(318) MRF2(340) CEBP(344) COREBINDINGFACTOR(344) OSF2(346) FXR(370) |
| 11 | chr11:6460846-6461263 | intron | 0.001 | chr11:6420962-6497746 | Ccm2 | 8 :: FOXO3(178) FOXO4(178) OSF2(210) AML(211) COREBINDINGFACTOR(212) PADS(213) PU1(225) TAL1BETAE47(262) |
| 12 | chr11:17112481-17112728 | promoter | 1.616 | chr11:17100398-17112862 | Pno1 | 5 :: HSF(16) IK3(41) APOLYA(78) PAX2(81) GR(82) |
| 13 | chr11:45664960-45665176 | promoter | 0.749 | chr11:44818751-45739501 | Clint1 | 1 :: TST1(156) |
| 14 | chr11:70818405-70818592 | promoter | 0.094 | chr11:70796538-70820942 | Dhx33 | 1 :: PLZF(126) |
| 15 | chr11:74466035-74467351 | intron | 0.003 | chr11:74455362-74486426 | 1300001I01Rik | 35 :: SREBP(231) EGR3(258) EBF(407) STAF(445) MYB(511) E47(514) OLF1(521) EBF(521) LYF1(524) USF(559) E4F1(588) STRA13(593) DR1(634) ZIC3(656) MYOGENIN(719) TAL1ALPHAE47(720) LMO2COM(720) AP4(720) TAL1(720) LBP1(720) RP58(721) AP4(732) MYOGENIN(732) LMO2COM(733) TAL1(733) LBP1(733) DEC(858) ZID(965) NRSF(1047) HSF(1050) AP2REP(1075) MEF3(1083) SMAD3(1203) NRSF(1241) MINI19(1287) |
| 16 | chr11:100180665-100180897 | promoter | 1.000 | chr11:100136694-100195706 | Eif1 | 2 :: OCT1(141) SREBP(188) |
| 17 | chr11:116705089-116705192 | promoter | 0.582 | chr11:116689361-116705242 | Jmjd6 | 3 :: E47(0) HIF1(0) AHRHIF(5) |
| 18 | chr12:40983796-40984415 | intergenic | 0.011 | chr12:40925967-41040688 | Ifrd1 | 6 :: CEBP(84) HSF2(148) AP3(305) NKX61(336) CEBP(555) ZIC1(583) |
| 19 | chr13:14993792-14994794 | intergenic | 0.971 | chr13:14718227-15555522 | AW209491 | 14 :: FOXJ2(69) OCT1(122) TBX5(181) GR(185) LEF1TCF1(188) STAT(233) PTF1BETA(256) PXR(281) MEIS1BHOXA9(345) OCT1(411) TBP(557) BRN2(632) CDPCR1(677) LEF1TCF1(920) |
| 20 | chr13:34106165-34106559 | intron | 0.956 | chr13:34082229-34129509 | Ripk1 | 4 :: PXR(36) PTF1BETA(150) SMAD3(183) TBX5(365) |
| 21 | chr14:8927355-8927809 | intergenic | 0.909 | chr14:8889081-8930753 | Rpp14 | 6 :: ZIC3(181) HSF(210) FOXM1(286) RP58(338) RFX1(341) CEBP(341) |
| 22 | chr14:27457350-27457469 | promoter | 0.002 | chr14:27447900-27483487 | Arf4 | 3 :: STAF(17) STAT3(68) STAF(112) |

| | | | | | | |
|---|---|---|---|---|---|---|
| 23 | chr14:32319804-32320354 | UTR3 | 0.005 | chr14:32308329-32337525 | Eaf1 | 3 :: NERF(20) OCT1(266) MYB(524) |
| 24 | chr15:10390832-10391259 | intergenic | 0.653 | chr15:10339652-10416820 | Bxdc2 | 10 :: STAT6(85) HSF(117) TAL1(170) LBP1(170) FOXO3(223) FOXO4(223) XFD3(223) BRN2(231) PAX2(314) ZIC1(346) |
| 25 | chr15:34011249-34011680 | promoter | 0.002 | chr15:33617656-34167620 | Mtdh | 3 :: CEBPGAMMA(73) CDPCR3(81) MRF2(228) |
| 26 | chr15:38923294-38923642 | intergenic | 1.097 | chr15:38918882-38944881 | Slc25a32 | 3 :: STRA13(58) E47(59) AML(247) |
| 27 | chr15:75754856-75754990 | intergenic | 1.832 | chr15:75746893-75755034 | Pycrl | 3 :: NERF(58) AP4(64) NERF(65) |
| 28 | chr15:76140352-76140509 | intergenic | 0.361 | chr15:76137701-76161634 | Exosc4 | 2 :: STAT(53) HSF2(68) |
| 29 | chr15:76276880-76277426 | intergenic | 0.005 | chr15:76205381-76308820 | Bop1 | 20 :: TBX5(69) E47(71) ZID(99) STAT6(181) LMO2COM(305) E47(340) TBX5(342) ROAZ(353) EBF(353) FOXD3(391) PAX2(395) MYOGENIN (428) AP4(429) LBP1(429) E47(430) TBX5(431) GR(466) TAL1ALPHAE47(469) TAL1BETAE47(469) TAL1(469) |
| 30 | chr15:76289677-76289991 | UTR3 | 2.192 | chr15:76286394-76289890 | Scx | 5 :: AP2REP(96) PAX(102) OCT1(160) OCT1(183) CEBP(184) |
| 31 | chr15:80083413-80083850 | intergenic | 0.291 | chr15:80045977-80085585 | Smcr7l | 10 :: GR(11) COUP(19) DR1(19) PPARA(22) MRF2(65) MYB(179) NKX25(194) STRA13(195) USF(195) AP1FJ(220) |
| 32 | chr15:101979552-101979610 | promoter | 0.215 | chr15:101979562-102007007 | Soat2 | 1 :: KAISO(14) |
| 33 | chr16:11219626-11220919 | intergenic | 0.057 | chr16:11176439-11220485 | Rsl1d1 | 11 :: AP3(211) HSF(362) STAT4(567) OCT1(628) BRN2(653) CDX(762) MSX1(783) RFX1(803) FOXO4(828) NKX61(849) MSX1(855) |
| 34 | chr16:16302744-16302863 | promoter | 1.016 | chr16:16272839-16312172 | Yars2 | 2 :: OCT1(34) AML(99) |
| 35 | chr16:16302991-16303076 | UTR5 | 0.319 | chr16:16272839-16312172 | Yars2 | 2 :: NRF1(11) STAF(65) |
| 36 | chr16:37390475-37391666 | intergenic | 1.022 | chr16:37385019-37540300 | Gtf2e1 | 11 :: COREBINDINGFACTOR(35) PAX(369) OCT1(623) TBP(663) AP3(768) TBP(797) XFD3(897) PADS(991) IRF1(996) CDX2(1016) RUSH1A(1018) |
| 37 | chr16:78377696-78377829 | promoter | 0.486 | chr16:78360101-78543749 | Btg3 | 2 :: BRACH(88) TBX5(91) |
| 38 | chr17:17539377-17539908 | intergenic | 1.714 | chr17:17286256-17539520 | Riok2 | 2 :: TST1(34) AP3(34) |
| 39 | chr17:46919582-46919669 | UTR5 | 0.549 | chr17:46910856-47027562 | Rpl7l1 | 2 :: NRF1(24) ZID(81) |
| 40 | chr17:94971998-94972853 | intergenic | 0.000 | chr17:94126646-95205589 | Mettl4 | 14 :: STAT(83) EBF(84) OCT1(214) PAX(275) LHX3(325) IPF1(427) OLF1(441) RFX1(482) FXR(490) STAT(509) FREAC3(512) IRF1(554) FOXO4(762) GR(765) |
| 41 | chr18:52676293-52677582 | intergenic | 0.547 | chr18:52492443-52676773 | Srfbp1 | 7 :: PAX2(154) CEBP(157) COUP(224) DR1(225) FOXO4(275) APOLYA(317) SMAD3(372) |
| 42 | chr19:16000265-16000545 | promoter | 0.006 | chr19:14983901-16029900 | Psat1 | 1 :: CEBP(104) |
| 43 | chr19:44123246-44123611 | intron | 0.003 | chr19:44105557-44144016 | Erlin1 | 3 :: BRN2(184) NKX25(216) ZTA(358) |
| 44 | chr19:47164544-47164741 | promoter | 0.706 | chr19:47163934-47206575 | Pdcd11 | 2 :: AP4(115) CDPCR3(132) |
| 45 | chr19:47204679-47205128 | intron | 0.584 | chr19:47163934-47206575 | Pdcd11 | 1 :: PAX(388) |
| 46 | chr2:22773771-22774117 | intron | 0.374 | chr2:22731361-22795779 | Pdss1 | 5 :: ROAZ(14) ATATA(97) NKX25(100) STAT6(115) MRF2(169) |

| | | | | | | |
|---|---|---|---|---|---|---|
| 47 | chr2:24829583-24829684 | UTR3 | 0.013 | chr2:24827990-24830618 | Mrpl41 | 1 :: APOLYA(72) |
| 48 | chr2:26748246-26748386 | intron | 0.132 | chr2:26721513-26758377 | Surf6 | 1 :: DELTAEF1(140) |
| 49 | chr2:32559294-32559576 | intergenic | 0.018 | chr2:32537680-32561077 | Fpgs | 2 :: AP1FJ(210) AP4(241) |
| 50 | chr2:72313092-72313239 | promoter | 0.319 | chr2:72280686-72774438 | Cdca7 | 1 :: CDPCR1(97) |
| 51 | chr2:72313269-72313408 | promoter | 0.700 | chr2:72280686-72774438 | Cdca7 | 3 :: RUSH1A(59) CEBP(64) CEBPGAMMA(67) |
| 52 | chr2:120392880-120392921 | promoter | 1.928 | chr2:120389575-120429403 | Snap23 | 3 :: PAX(19) HSF(20) STAF(26) |
| 53 | chr2:130437095-130437624 | intron | 0.003 | chr2:130408332-130458554 | Fastkd5, Ubox5 | 6 :: ZEC(51) PAX3(134) XBP1(303) AP3(420) FOXM1(424) AIRE(437) |
| 54 | chr2:132631832-132632344 | intron | 1.258 | chr2:132621676-132642996 | Trmt6 | 8 :: OLF1(45) E47(88) LMO2COM(88) AP4(88) TAL1(88) LBP1(88) MYOGENIN(89) OCT1(395) |
| 55 | chr2:132642271-132642833 | promoter | 1.341 | chr2:132621676-132642996 | Trmt6 | 3 :: ZEC(34) CEBP(120) AIRE(295) |
| 56 | chr3:37372064-37372873 | intron | 0.009 | chr3:37318256-37538845 | Spata5 | 7 :: STAT4(222) STAT6(222) CEBPDELTA(337) CDX(689) NKX25(694) IRF1(713) ATATA(773) |
| 57 | chr3:87856041-87856299 | intron | 0.165 | chr3:87831436-87860375 | Gpatch4 | 2 :: AHRARNT(113) TAL1(179) |
| 58 | chr3:122079008-122079817 | intergenic | 0.012 | chr3:121969592-122122744 | Dnttip2 | 10 :: R(111) AP4(113) KAISO(201) VJUN(434) ATF3(434) CREBATF(435) SPZ1(510) PU1(540) PAX(618) AP1FJ(733) |
| 59 | chr3:137590985-137591878 | intergenic | 0.163 | chr3:137591779-137708137 | Dapp1 | 2 :: NKX25(797) TFE(815) |
| 60 | chr4:45071170-45072087 | intergenic | 0.001 | chr4:45025172-45117976 | Polr1e | 27 :: STAT3(137) STAT(140) NRF1(150) MYOGENIN(240) AP4(241) LBP1(242) MYOGENIN(248) LBP1(249) STAT(283) HIF1(306) EGR3(307) AHRHIF(307) CDPCR1(497) ZTA(507) USF(512) R(529) FXR(559) MEIS1BHOXA9(564) FOXO4(680) MEIS1BHOXA9(704) HIF1(725) AHRHIF(725) CEBP(753) IRF1(770) DEC(771) USF(771) MYB(821) |
| 61 | chr4:48291304-48291574 | promoter | 1.387 | chr4:48291405-48444825 | Invs | 2 :: NKX61(107) AP3(157) |
| 62 | chr4:48480777-48481152 | intron | 0.832 | chr4:48443828-48553346 | Tex10 | 3 :: IRF1(135) PLZF(171) PLZF(189) |
| 63 | chr4:108510642-108511225 | intron | 0.664 | chr4:108505790-108551674 | Txndc12 | 3 :: EGR3(98) DR1(283) MEIS1BHOXA9(507) |
| 64 | chr4:132048769-132049475 | UTR3 | 0.006 | chr4:132024048-132086198 | Sesn2 | 16 :: PPARA(1) OLF1(158) SREBP(205) CDPCR3(214) DR1(242) LDSPOLYA(323) E47(333) TAL1ALPHAE47(333) TAL1BETAE47(333) AP4(333) LBP1(333) CDPCR1(356) NFKAPPAB65(486) STAT3(487) STAT6(500) PU1(568) |
| 65 | chr5:20492445-20493250 | UTR3 | 0.010 | chr5:20457641-20688227 | Ptpn12 | 31 :: FOXD3(34) FOXJ2(35) FOXO4(37) HNF3ALPHA(37) FOXO3(38) RFX1(158) HIF1(162) MSX1(165) DR3(167) FOXO3(247) FOXO4(247) XFD3(247) FOXJ2(266) LEF1TCF1(284) LMO2COM(293) ZEC(306) MINI19(314) COUP(330) ATATA(454) FREAC3(473) PAX2(494) PXR(586) IPF1(595) CDX2(605) FOXJ2(606) PAX2(609) TTF1(610) CIZ(640) NFAT(643) IK3(674) FOXJ2(686) |
| 66 | chr5:36963855-36964094 | UTR5 | 0.713 | chr5:36925023-37138549 | D5Ertd579e | 3 :: DELTAEF1(10) TBX5(11) E4F1(109) |
| 67 | chr5:77739571-77739720 | promoter | 0.317 | chr5:77712075-77739835 | 2610024G14Rik | 2 :: AHRHIF(104) PAX3(111) |
| 68 | chr5:115791159-115791183 | promoter | 1.697 | chr5:115790745-115793561 | Triap1 | 1 :: CHCH(18) |
| 69 | chr5:115791202-115791279 | UTR5 | 0.499 | chr5:115790745-115793561 | Triap1 | 5 :: NRF1(2) PAX3(30) VJUN(34) ATF3(34) CREBATF(34) |

| | | | | | | |
|---|---|---|---|---|---|---|
| 70 | chr5:115793402-115793614 | UTR3 | 2.440 | chr5:115790745-115793561 | Triap1 | 2 :: FOXO3(98) HNF3ALPHA(101) |
| 71 | chr5:116475339-116475501 | promoter | 0.482 | chr5:116456395-116575565 | Prkab1 | 1 :: CDX2(138) |
| 72 | chr5:121889960-121890352 | UTR3 | 0.044 | chr5:121836622-121893925 | C330023M02Rik | 11 :: RP58(72) TAL1ALPHAE47(73) TAL1BETAE47(73) PU1(103) DR3(116) PAX3(116) TCF4(125) LEF1TCF1(127) PAX3(132) ATATA(183) TBP(225) |
| 73 | chr5:136407952-136408618 | intron | 0.005 | chr5:136367448-136455806 | Ywhag | 17 :: CEBPGAMMA(234) TAL1(406) MYOGENIN(407) LMO2COM(414) CEBPDELTA(426) DELTAEF1(476) AP1FJ(494) ZTA(497) VJUN(508) ATF3(508) CREBATF(509) PADS(510) AIRE(519) RFX1(523) CDPCR3(528) TBX5(534) AHRARNT(544) |
| 74 | chr5:147759258-147759445 | promoter | 0.831 | chr5:147659307-147767179 | Gtf3a | 2 :: NRF1(2) POLY(114) |
| 75 | chr5:147807260-147807905 | intergenic | 2.005 | chr5:147766098-147828678 | Mtif3 | 12 :: TTF1(195) CDX(252) CEBPDELTA(253) VBP(253) OCT1(254) TBP(286) SMAD3(310) CEBPGAMMA(323) STAF(394) STAT6(474) NFKAPPAB65(536) SREBP(613) |
| 76 | chr5:150048769-150049550 | intergenic | 3.571 | chr5:149864627-150076628 | Uspl1 | 11 :: AP4(198) LBP1(198) VJUN(288) ATF3(288) CREBATF(288) OCT1(362) NKX25(405) MEIS1BHOXA9(444) ZEC(572) AP4(663) LDSPOLYA(728) |
| 77 | chr6:58468540-58469245 | intergenic | 0.050 | chr6:58387614-58783636 | Abcg2 | 5 :: SMAD3(141) TBP(153) MSX1(224) DEC(330) LYF1(334) |
| 78 | chr7:3592078-3592336 | intron | 0.868 | chr7:3580377-3596911 | Prpf31 | 2 :: AP2REP(162) LYF1(179) |
| 79 | chr7:35829530-35830027 | intergenic | 0.388 | chr7:35829741-35904302 | Cebpg | 4 :: PU1(214) STRA13(428) USF(428) CEBP(480) |
| 80 | chr7:35832900-35833380 | UTR3 | 0.306 | chr7:35829741-35904302 | Cebpg | 6 :: EBF(71) COUP(149) CDX(250) AIRE(257) NKX61(261) GR(386) |
| 81 | chr7:71517584-71518479 | intergenic | 0.017 | chr7:71487048-71538616 | Mphosph10 | 15 :: TBX5(50) SREBP(52) TAL1BETAE47(118) STAT3(343) LYF1(346) TBX5(502) MYB(682) MYOGENIN(689) E47(690) TAL1ALPHAE47(690) LMO2COM(690) TAL1(690) USF(690) AP4(691) CREBATF(800) |
| 82 | chr7:105850380-105851066 | promoter | 0.009 | chr7:105805097-105987342 | Prkrir | 14 :: CEBPDELTA(60) PAX(81) TCF4(147) MYB(314) FXR(334) PXR(337) STAF(415) RUSH1A(427) AIRE(431) ZIC1(495) XBP1(512) OCT1(533) LEF1TCF1(543) GR(543) |
| 83 | chr8:47620772-47621477 | UTR3 | 0.009 | chr8:47382700-47636745 | Acsl1 | 24 :: STAT3(181) STAT4(219) PAX(250) IRF1(254) AIRE(283) CEBP(288) LYF1(290) PTF1BETA(295) IRF1(308) CEBP(328) NKX25(484) BRACH(516) FOXD3(529) FOXJ2(530) HNF3ALPHA(532) GR(575) NFAT(578) OCT1(580) FOXO4(598) HNF3ALPHA(605) FOXJ2(606) NKX62(606) FOXD3(607) CDPCR3(609) |
| 84 | chr8:48618537-48618767 | promoter | 0.645 | chr8:48617884-48752204 | Rwdd4a | 7 :: HIF1(145) AHRHIF(145) AP4(158) LMO2COM(164) AP4(164) LBP1(164) SPZ1(216) |
| 85 | chr8:48712003-48712778 | intergenic | 0.696 | chr8:48617884-48752204 | Rwdd4a | 9 :: STAT4(38) OCT1(49) CEBPGAMMA(66) OCT1(72) BRACH(125) CEBP(425) MEF3(486) LYF1(533) NFKAPPAB65(662) |
| 86 | chr8:109456116-109456760 | intergenic | 0.006 | chr8:109417494-109459642 | Cirh1a | 15 :: CDX(58) VBP(66) VBP(83) STAT3(161) IK3(163) MEIS1BHOXA9(177) AP4(268) LBP1(269) E47(367) LMO2COM(367) HSF(392) NFAT(472) NFKAPPAB65(474) TFE(611) DEC(612) |
| 87 | chr8:113573352-113573598 | intron | 0.170 | chr8:113551672-113593195 | Aars, Exosc6 | 2 :: XBP1(102) HIF1(102) |
| 88 | chr8:126520131-126520512 | UTR3 | 1.332 | chr8:126507040-126545209 | Taf5l | 8 :: PLZF(102) FOXO3(111) FOXO4(111) XFD3(111) MRF2(116) CEBPGAMMA(134) NRF1(202) COREBINDINGFACTOR(289) |
| 89 | chr9:35017379-35017529 | promoter | 0.600 | chr9:35017433-35024588 | Srpr | 3 :: COUP(70) COUP(77) ATF3(145) |

| | | | | | | |
|---|---|---|---|---|---|---|
| **90** | chr9:107899506-107900141 | intergenic | 3.805 | chr9:107876167-107904976 | D330022A01Rik, Ube1l | 15 :: NERF(80) FXR(83) CHCH(96) ROAZ(99) PAX(122) AP1FJ(163) TTF1(187) STAF(219) EGR3(223) STAT3(313) STAT(313) IK3(315) NRSF(509) PAX3(584) DELTAEF1(607) |
| **91** | chr9:109798990-109799748 | intergenic | 0.003 | chr9:109751964-109833781 | Cdc25a | 13 :: DR3(36) NKX25(104) LBP1(199) MYOGENIN(200) STAF(201) AP4(266) STRA13(390) MYOGENIN(391) RP58(418) RFX1(462) TAL1BETAE47(556) NFAT(681) TBP(714) |
| **92** | chr9:119235322-119235640 | intergenic | 0.809 | chr9:119213978-119250657 | Myd88 | 3 :: OCT1(205) PADS(300) CREBATF(301) |
| **93** | chrX:72341166-72341266 | UTR5 | 1.812 | chrX:72330260-72355109 | Dkc1 | 2 :: NRF1(20) CHCH(99) |
| **94** | chrX:72354135-72354354 | UTR3 | 0.127 | chrX:72330260-72355109 | Dkc1 | 2 :: ATF3(99) SREBP(118) |
| **95** | chrX:131071648-131072128 | UTR3 | 0.222 | chrX:131011822-131077499 | Timm8a1 | 3 :: HSF(40) MSX1(190) OCT1(345) |
| **96** | chrX:131077064-131077274 | promoter | 0.164 | chrX:131011822-131077499 | Timm8a1 | 5 :: COUP(32) DR1(32) IK3(75) IK3(140) PTF1BETA(144) |

# CLUSTER 1 EPO RESPONSE GENES

| # | Regulatory element | Type | Score | Locus | Gene | Candidate transcription factor binding sites (relative positions) |
|---|---|---|---|---|---|---|
| 1 | chr1:9536703-9537191 | UTR3 | 2.856 | chr1:9289982-9538156 | Rrs1 | 5 :: ATF3(77) MOVOB(327) CHCH(327) MIF1(436) RFX1(437) |
| 2 | chr1:37799603-37800356 | intergenic | 1.341 | chr1:37496193-37811598 | Mgat4a | 8 :: SOX(88) SMAD3(251) TCF4(256) LEF1TCF1(258) BRN2(306) SRF(335) PAX3(672) IRF7(693) |
| 3 | chr1:88191006-88191258 | intergenic | 0.006 | chr1:88175483-88240366 | B3gnt7 | 4 :: E2F(74) STAF(105) SMAD3(138) CACCCBINDINGFACTOR(225) |
| 4 | chr1:121421987-121422143 | promoter | 0.310 | chr1:121401216-121444578 | Tmem185b | 4 :: PXR(60) COUP(66) COUP_DR1(66) PPAR_DR1(66) |
| 5 | chr1:121444042-121444407 | intergenic | 0.780 | chr1:121401216-121444578 | Tmem185b | 2 :: TAXCREB(95) SMAD3(201) |
| 6 | chr1:182834306-182834438 | promoter | 1.410 | chr1:182829946-182865130 | Pycr2 | 11 :: E2F(26) NRF1(28) MYCMAX(32) ZF5(32) ALPHACP1(83) EBOX(97) MYCMAX(98) XBP1(98) MAX(98) MYC(98) USF2(98) |
| 7 | chr10:76446303-76446679 | intergenic | 1.035 | chr10:76424547-76514664 | Slc19a1 | 6 :: IRF1(72) FXR_IR1(100) AR(151) GRE(151) SF1(156) MYOGENIN(165) |
| 8 | chr10:76446933-76447294 | intergenic | 0.867 | chr10:76424547-76514664 | Slc19a1 | 3 :: HMX1(83) LDSPOLYA(103) NRF1(126) |
| 9 | chr10:94799305-94800027 | intergenic | 2.425 | chr10:94786738-94943373 | Socs2 | 14 :: GATA1(13) STAT5B(156) PAX6(204) GRE(340) AR(341) SOX9_B1(343) SOX(344) OCT(361) COUP_DR1(385) PPAR_DR1(385) NFKAPPAB(397) XBP1(420) FXR(626) ATF3(682) |
| 10 | chr10:94880514-94880825 | promoter | 0.363 | chr10:94786738-94943373 | Socs2 | 13 :: STAT1(25) STAT5B(60) CEBPB(79) COUP(99) ERR1(101) MEIS1AHOXA9(103) FXR(105) PXR(105) COUP(106) COUP_DR1(107) FXR_IR1(107) PPAR_DR1(107) E2F(178) |
| 11 | chr11:48103820-48104697 | intergenic | 0.001 | chr11:47614030-48619743 | Gnb2l1 | 28 :: E2(33) RFX1(43) NFE2(43) GRE(345) COUP(360) COUP(392) CDX2(398) FXR(398) FXR_IR1(400) PXR(401) AP3(412) FXR(506) ERR1(552) AR(556) FXR(556) FXR_IR1(558) NKX3A(562) COUP(577) SF1(580) SOX(589) OTX(604) XVENT1(678) S8(731) LHX3(731) AR(755) RBPJK(809) CART1(810) COUP(863) |
| 12 | chr11:74714303-74714604 | intron | 0.292 | chr11:74710083-74722842 | Tsr1 | 1 :: TBP(69) |
| 13 | chr11:74740337-74740446 | promoter | 0.475 | chr11:74720298-74740704 | Srr | 2 :: GATA1(45) FAC1(105) |
| 14 | chr11:101319312-101319414 | intron | 0.345 | chr11:101306849-101320015 | Ifi35 | 2 :: GC(11) CACBINDINGPROTEIN(16) |
| 15 | chr11:109334985-109335258 | promoter | 0.005 | chr11:109300303-109351136 | Slc16a6 | 19 :: ZF5(30) E2F(30) ZF5(49) E2F(51) E2(87) TBX5(93) NRF1(105) MYCMAX(107) ZF5(109) E2F(109) STAT1(142) STAT3(142) TAXCREB(160) ZF5(167) STAT1(180) ISRE(184) STAT1(190) ZF5(231) MOVOB(268) |
| 16 | chr11:120319593-120319724 | intron | 0.633 | chr11:120317988-120325667 | Ccdc137 | 3 :: R(112) ATF3(123) ER(126) |
| 17 | chr12:72238797-72239069 | promoter | 0.005 | chr12:72200350-72410919 | Timm9 | 2 :: POLY(183) AR(223) |
| 18 | chr12:80051069-80051774 | intergenic | 0.009 | chr12:79988351-80061217 | Plek2 | 7 :: ALPHACP1(220) PXR(280) AR(326) S8(360) RFX1(378) ROAZ(422) AR(487) |
| 19 | chr12:88729421-88730015 | intergenic | 1.154 | chr12:88729183-88786353 | Alkbh1, Nrp | 10 :: GC(64) GC(103) S8(125) ALPHACP1(125) PAX3(134) SMAD3(258) ELK1(260) MYCMAX(374) TEL2(522) PAX6(528) |
| 20 | chr12:88788111-88788326 | intron | 0.002 | chr12:88783431-88790868 | 1810035L17Rik | 3 :: PAX9(46) CEBPB(152) STAT1(154) |
| 21 | chr13:49557129-49558444 | UTR3 | 0.002 | chr13:49482430-49600173 | Ippk | 20 :: MIF1(18) BRCA(60) BRCA(65) TITF1(361) PAX(420) NFKAPPAB65(421) NFKAPPAB(421) LBP1(429) ER(616) PADS(627) NRF1(636) GC(733) AP1FJ(964) TBP(1059) OCT(1129) BRN2(1143) RBPJK(1181) STAT1(1184) NKX3A(1211) TITF1(1212) |
| 22 | chr13:49741889-49742248 | intergenic | 0.400 | chr13:49720078-49776010 | Nol8 | 3 :: LDSPOLYA(141) PLZF(207) FREAC2(208) |

| | | | | | | |
|---|---|---|---|---|---|---|
| 23 | chr13:49748133-49748246 | promoter | 1.178 | chr13:49720078-49776010 | Nol8 | 5 :: MYCMAX(12) NRF1(12) E2F(12) ZF5(13) TAXCREB(76) |
| 24 | chr13:107750499-107752100 | intergenic | 0.006 | chr13:107727008-107751294 | Dimt1 | 13 :: ERR1(31) SF1(32) FOXJ2(76) XVENT1(84) SRY(131) NKX3A(194) AP3(214) XFD3(235) RBPJK(422) STAT1(434) PAX6(732) IPF1(779) XBP1(787) |
| 25 | chr14:25927428-25928934 | intergenic | 0.002 | chr14:25306269-26278775 | Rps24 | 59 :: SRY(85) PAX2(154) STAF(168) CEBPB(173) ISRE(180) IRF1(180) CART1(188) S8(192) NKX62(192) STAT1(200) STAT3(200) STAT(200) STAF(205) BRN2(211) AP1FJ(218) NFE2(219) GATA1(226) IPF1(286) FXR_IR1(286) MTF1(317) FAC1(322) BRCA(324) XVENT1(410) E2F1DP2(413) ELK1(450) TEL2(452) NERF(453) ETS1(454) STAT1(482) TBX5(704) AFP1(829) IPF1(832) LHX3(832) AR(866) ALPHACP1 (886) SOX(887) P53_DECAMER(903) NRSE(936) CART1(964) GZF1(974) PAX3(1052) SRY(1061) FAC1(1064) SRY(1066) FXR(1089) PXR (1089) IPF1(1091) TCF11(1093) ZIC1(1106) TAXCREB(1108) RFX1(1111) E2F(1126) PAX6(1147) AP1FJ(1156) ATF3(1182) ER(1185) AR (1188) SOX(1207) MIF1(1366) |
| 26 | chr14:31829127-31829507 | intron | 0.003 | chr14:31825219-31833534 | Gnl3 | 3 :: PAX2(32) FOXP3(58) ATF3(276) |
| 27 | chr14:41720265-41720881 | UTR3 | 0.002 | chr14:41706263-41804940 | Tspan14 | 6 :: COUP(130) R(180) AR(365) TBP(377) HMEF2(378) IRF1(539) |
| 28 | chr14:51407495-51407890 | intergenic | 0.275 | chr14:51405196-51427614 | ##Ccnb1ip1, NM_001111119 | 5 :: MTF1(77) NFKAPPAB(112) CACCCBINDINGFACTOR(261) SF1(298) LEF1TCF1(308) |
| 29 | chr15:38923294-38923642 | intergenic | 0.747 | chr15:38918882-38944881 | Slc25a32 | 4 :: ER(61) AML(247) PEBP(247) MIF1(274) |
| 30 | chr15:80084541-80084655 | promoter | 0.412 | chr15:80083247-80090988 | Atf4 | 1 :: ETS1(80) |
| 31 | chr15:88692153-88692317 | promoter | 0.003 | chr15:88657115-88744210 | Pim3 | 8 :: CACBINDINGPROTEIN(67) EBOX(112) MYCMAX(113) MAX(113) MYC(113) USF2(113) NRF1(115) NRF1(131) |
| 32 | chr16:20611503-20611697 | UTR5 | 0.580 | chr16:20610271-20651772 | Ece2 | 9 :: ELK1(71) NFKAPPAB65(74) MYCMAX(81) XBP1(81) MAX(81) MYC(81) USF2(81) EBOX(82) GC(127) |
| 33 | chr16:78577585-78577740 | promoter | 0.404 | chr16:78377036-78931177 | D16Ertd472e | 3 :: ISRE(54) FXR_IR1(91) MIF1(112) |
| 34 | chr17:29270409-29270900 | intergenic | 0.021 | chr17:29183067-29271823 | Cdkn1a | 10 :: PAX9(31) SF1(117) E2F(185) R(217) COUP(436) COUP_DR1(436) PPAR_DR1(436) COUP(443) COUP_DR1(443) PPAR_DR1(443) |
| 35 | chr17:32153431-32154122 | intergenic | 1.871 | chr17:31992862-32257795 | Rrp1b | 9 :: SRY(20) ALPHACP1(46) BRCA(209) GRE(366) E2(489) ELK1(559) TBP(575) GRE(585) MYCMAX(661) |
| 36 | chr17:47748716-47748830 | promoter | 0.818 | chr17:47737029-47749929 | Bysl | 3 :: ER(11) TAXCREB(43) P53_DECAMER(90) |
| 37 | chr18:46756637-46757014 | promoter | 0.005 | chr18:46734195-46872283 | Eif1a | 15 :: STAT5B(10) PAX(14) R(98) PAX6(109) PAX(113) ROAZ(117) MTF1(120) NRSE(196) ZF5(258) PAX9(272) R(272) ZF5(292) E2F(344) MYCMAX(346) ZF5(346) |
| 38 | chr19:5730139-5730368 | UTR3 | 0.003 | chr19:5729654-5740903 | Sssca1 | 4 :: NRF1(135) CIZ(196) FOXP3(198) ALX4(199) |
| 39 | chr19:11018739-11019056 | intron | 1.204 | chr19:11017050-11032547 | Ccdc86 | 4 :: COUP(103) COUP_DR1(103) PPAR_DR1(103) CACBINDINGPROTEIN(128) |
| 40 | chr19:11031781-11032129 | intergenic | 1.294 | chr19:11017050-11032547 | Ccdc86 | 2 :: BRN2(312) HMEF2(314) |
| 41 | chr19:37507776-37508076 | promoter | 0.006 | chr19:37498390-37624901 | Hhex | 4 :: SMAD3(81) IPF1(86) TBX5(244) PAX6(263) |
| 42 | chr19:42008597-42008897 | promoter | 0.009 | chr19:41993180-42009271 | Exosc1 | 5 :: TCF4(105) LEF1TCF1(107) TAL1BETAE47(188) TAL1BETAITF2(188) GATA1(228) |
| 43 | chr19:45079821-45080087 | promoter | 0.534 | chr19:45079922-45095872 | Peo1 | 5 :: IPF1(106) ERR1(210) SF1(211) FXR(214) ER(214) |

| | | | | | | |
|---|---|---|---|---|---|---|
| 44 | chr2:10644876-10645895 | intergenic | 0.005 | chr2:10516791-11392901 | Prkcq | 13 :: RFX1(260) FOXM1(426) PXR(501) ER(709) PAX3(717) AP1FJ(721) MIF1(762) TCF11(771) IPF1(772) STAT5B(848) HLF(909) VBP(909) CEBPB(1015) |
| 45 | chr3:69408456-69408959 | intergenic | 0.194 | chr3:69402786-69622974 | Nmd3 | 5 :: FXR_IR1(105) RFX1(107) GATA1(108) MYOGENIN(235) LBP1(236) |
| 46 | chr3:84754820-84755451 | promoter | 0.006 | chr3:84509222-85378049 | Fbxw7 | 8 :: FREAC2(190) FREAC2(200) XFD3(201) SOX(340) LHX3(370) AFP1(376) HLF(529) VBP(529) |
| 47 | chr3:84769942-84770441 | promoter | 1.883 | chr3:84768978-84770799 | Dear1 | 3 :: HMEF2(323) HMEF2(331) FXR(484) |
| 48 | chr4:107473469-107473651 | promoter | 1.988 | chr4:107307792-107552389 | Lrp8 | 3 :: SMAD3(55) AML(144) PEBP(144) |
| 49 | chr4:124532075-124532775 | UTR3 | 1.114 | chr4:124524945-124532483 | Yrdc | 4 :: ELK1(80) S8(348) NKX3A(349) NKX62(350) |
| 50 | chr4:147976510-147976694 | intergenic | 0.009 | chr4:147961017-147976650 | Srm | 6 :: STAT3(94) STAT5B(94) STAT(94) STAF(98) GC(116) HNF3(134) |
| 51 | chr4:149149810-149149915 | promoter | 0.004 | chr4:149112156-149270372 | Slc25a33 | 3 :: TFIII(30) ZNF219(32) PAX6(65) |
| 52 | chr4:151412496-151412792 | promoter | 0.518 | chr4:151412294-151470821 | Nol9 | 10 :: MYOGENIN(57) LBP1(58) CACBINDINGPROTEIN(97) GC(98) EBOX(190) MAX(191) MYCMAX(191) AML(226) EBOX(252) MYOGENIN(252) |
| 53 | chr4:151413116-151413222 | promoter | 0.138 | chr4:151412294-151470821 | Nol9 | 2 :: SRY(9) ELK1(87) |
| 54 | chr5:147606844-147607074 | promoter | 0.661 | chr5:147396902-147644718 | Usp12 | 3 :: HMX1(88) FXR_IR1(112) S8(146) |
| 55 | chr5:149138945-149139630 | UTR3 | 0.002 | chr5:149125669-149316194 | Slc7a1 | 10 :: TITF1(23) PADS(29) PAX3(82) GRE(154) XVENT1(348) LDSPOLYA(398) PADS(472) AML(474) PEBP(474) PAX2(543) |
| 56 | chr6:82792732-82793336 | intergenic | 0.299 | chr6:82602868-82861427 | Hk2 | 10 :: ALPHACP1(35) XVENT1(57) ELK1(110) NERF(113) LBP1(123) MYOGENIN(123) ELK1(239) ETS1(243) NFE2(414) TAXCREB(415) |
| 57 | chr6:83194538-83195250 | intergenic | 0.003 | chr6:83150447-83274714 | Mthfd2 | 18 :: STAF(90) SOX(175) NKX3A(213) ETS1(231) NFKAPPAB(289) E2F(309) AP1FJ(353) AR(426) FOXM1(484) PLZF(485) IPF1(493) ER(554) SF1(556) ERR1(557) ZIC1(595) AR(615) FXR_IR1(687) ROAZ(710) |
| 58 | chr6:91415780-91416198 | intron | 0.005 | chr6:91361365-91424899 | Chchd4 | 4 :: XBP1(62) MYCMAX(62) SRF(339) STAF(382) |
| 59 | chr6:122563365-122563467 | intergenic | 0.005 | chr6:122553354-122575228 | Gdf3 | 6 :: ER(29) LEF1TCF1(95) COUP(96) TCF4(96) COUP_DR1(97) PPAR_DR1(97) |
| 60 | chr6:127078420-127079466 | UTR3 | 0.685 | chr6:127059565-127403705 | Ccnd2 | 27 :: LUN1(82) CDX2(108) FOXJ2(114) GZF1(142) GRE(149) E2F(215) HNF3(371) SOX(391) CEBPB(423) STAT(505) E2(584) STAT1(589) E2 (590) TBX5(608) CACCCBINDINGFACTOR(612) FOXP3(638) TAL1BETAITF2(717) NERF(853) E2F1DP2(862) OCT(892) TCF4(938) BRN2(948) SOX(968) P53(991) OCT(995) GATA1(997) E2F(1003) |
| 61 | chr6:149233644-149234451 | intergenic | 3.846 | chr6:149104465-149357565 | 2810474O19Rik | 11 :: SF1(153) R(186) RUSH1A(241) IRF1(249) PAX3(268) RUSH1A(418) XFD3(528) NKX3A(626) NKX3A(634) PXR(651) CDX2(663) |
| 62 | chr6:149334681-149335490 | intergenic | 3.753 | chr6:149104465-149357565 | 2810474O19Rik | 9 :: CART1(266) IPF1(270) SF1(318) FXR(321) ELK1(445) LDSPOLYA(528) PAX3(561) MEIS1AHOXA9(568) FOXM1(647) |

| | | | | | | |
|---|---|---|---|---|---|---|
| 63 | chr8:87225071-87225171 | promoter | 0.008 | chr8:87223710-87231462 | Lyl1 | 1 :: SF1(80) |
| 64 | chr8:96909439-96909705 | promoter | 0.010 | chr8:96890011-97048954 | Herpud1 | 7 :: ER(85) ERR1(88) LUN1(132) IRF7(169) ISRE(170) IRF1(170) SRY(170) |
| 65 | chr8:109582397-109582507 | UTR3 | 0.357 | chr8:109579607-109584427 | Nip7 | 1 :: SRY(46) |
| 66 | chr9:34998670-34999185 | UTR5 | 0.076 | chr9:34983576-35007459 | Tirap | 5 :: PAX3(222) EBOX(275) MYOGENIN(275) R(280) ELK1(342) |
| 67 | chr9:99012943-99013576 | intron | 0.429 | chr9:98938735-99142681 | Pik3cb | 7 :: TBX5(133) GATA1(164) TAL1BETAE47(165) TAL1BETAITF2(165) OCT(358) IRF1(371) PAX2(447) |
| 68 | chr9:99040833-99041091 | promoter | 0.161 | chr9:98938735-99142681 | Pik3cb | 2 :: LBP1(112) MYOGENIN(113) |
| 69 | chr9:103257644-103258343 | UTR3 | 0.007 | chr9:103255432-103326993 | Cdv3 | 22 :: STAT1(7) OTX(17) FOXJ2(25) POLY(29) ALX4(118) RUSH1A(156) PAX6(175) OTX(426) GFI1B(459) STAF(474) P53(506) P53_DECAMER (506) MYOGENIN(535) LBP1(536) AR(541) PADS(542) R(547) CACBINDINGPROTEIN(562) RBPJK(623) XBP1(666) SRF(672) TCF11(684) |
| 70 | chr9:109798990-109799748 | intergenic | 0.366 | chr9:109751964-109833781 | Cdc25a | 12 :: LBP1(199) MYOGENIN(200) STAF(201) P53(211) EBOX(391) MYOGENIN(391) CACCCBINDINGFACTOR(421) MIF1(461) RFX1(462) TAL1BETAE47(556) TAL1BETAITF2(556) TBP(714) |
| 71 | chr9:114658149-114658503 | UTR3 | 2.830 | chr9:114633042-114665801 | Cmtm6 | 5 :: TBP(80) CDX2(143) NKX62(149) HMEF2(294) POLY(301) |
| 72 | chrX:53983994-53984142 | promoter | 0.054 | chrX:53915090-54050873 | Fhl1 | 2 :: HNF3(85) MTF1(137) |
