# Supplementary material for: Defining an EPOR- Regulated Transcriptome for Primary Progenitors, including Tnfr-sf13c as a Novel Mediator of EPO- Dependent Erythroblast Formation
Source: PLoS One. 2012 Jul 13;7(7):e38530. doi: 10.1371/journal.pone.0038530 (PMC3396641; doi:10.1371/journal.pone.0038530)
Supplement: Table S3 — Epo Modulated Transcription Factors. (PDF) [file pone.0038530.s007.pdf]

SUPPLEMENTAL TABLE S3: EPO/EPOR MODULATED TRANSCRIPTION FACTORS

gene symbol, gene name [Entrez gene ID]	EPO modulation, fold change	known / novel	description	reference (PMID #)
Klf3, Kruppel-like factor 3 (basic)[16599]	2.4x down	N	Binds to the CACCC box of erythroid cell-expressed genes; may play a role in hematopoiesis	17283065, 10582345
Mllt3, myeloid/lymphoid or mixed-lineage leukemia; translocated to, 3 [70122]	3.3x down	N	Regulates early human erythroid and megakaryocytic cell fate	18371451
Taf4b, TAF4B RNA polymerase II, TBP-associated factor [72504]	2.8x up	N	Cell type-specific subunit of TFIID that may function as a gene-selective coactivator in certain cells	16205117, 11856754
Atf4, activating transcription factor 4 [11911]	2.6x up	N	Transcriptional activator; binds the cAMP response element (CRE), a sequence present in many viral and cellular promoters	20592469, 17466566
Tsc22d1,TSC22 domain family, member 1 [21807]	2.6x down	N	Transcriptional repressor; acts on the C-type natriuretic peptide (CNP) promoter	20802130, 17690703
Sertad3, SERTA domain containing 3 [170742]	2.2x down	N	Strong transcriptional coactivator	10982866, 17260023
Hbp1, high mobility group box transcription factor 1 [73389]	2.2x down	N	Transcriptional repressor that binds to the promoter region of target genes	20581871
Fam117a, family with sequence similarity 117, memberA [215512]	2.2x down	N	Marker for FecL locus influencing ovulation rate in Lacaune sheep	19466934
Jmjd1c, jumonji domain containing 1C [108829]	2.1x down	N	histone demethylase; transcriptionally regulate mouse testis development	20530532
