# Supplementary material for: Defining an EPOR- Regulated Transcriptome for Primary Progenitors, including Tnfr-sf13c as a Novel Mediator of EPO- Dependent Erythroblast Formation
Source: PLoS One. 2012 Jul 13;7(7):e38530. doi: 10.1371/journal.pone.0038530 (PMC3396641; doi:10.1371/journal.pone.0038530)
Supplement: Table S4 — Epo/Epor Modulated Metabolism. (PDF) [file pone.0038530.s008.pdf]

SUPPLEMENTAL TABLE S4: EPO/EPOR MODULATED METABOLISM

Ubiquitination

gene symbol, gene name [Entrez gene ID]	EPO modulation, fold change	known / novel	description	reference (PMID #)
Herpud1, homocysteine-inducible, endoplasmic reticulum stress-inducible, ubiquitin-like domain member 1 [64209]	2.3x up	N	degrades misfolded ER proteins in ubiquitin-dependent manner	12370023, 12370023
Usp12, ubiquitin specific peptidase 12 [22217]	2.5x up	N	deubiquitinating enzyme, needs WDR48 for high activity	21183687, 19075014
Fbxw7, F-box and WD-40 domain protein 7 [50754]	3.0x up	N	substrate recognition component of SCF ubiquitin ligase and involved in degradation of factors that function in cellular growth and division pathways like cyclin-E and MYC	18094723, 18948752
Rnf103, ring finger protein 103 [22644]	2.0x down	N	E2-dependent E3 ubiquitin -protein ligase, involved in ER-associated protein degradation pathway	18615712, 18675248
N4bp2l1, NEDD4 binding protein 2-like 1 [100637]	3.3x down	N	N4bp2 can bind to both BCL-3 and Nedd4, can be an ubiquitylation substrate, may have role in DNA repair or recombination	176266400

Post-translational Modification

gene symbol, gene name [Entrez gene ID]	EPO modulation, fold change	known / novel	description	reference (PMID #)
B3gnt7, UDP-GlcNAc:betaGal beta-1,3-N-acetylglucosaminyltransferase 7 [227327]	2.3x up	N	prevent cells from migrating out of original tissues and invading surrounding tissues, biosynthesis of keratane sulfate	12061784, 17690104
Mgat4a, mannoside acetylglucosaminyltransferase 4, isoenzyme A [269181]	3.1x up	N	essential for production of tri- and tetra-antennary N-linked sugar chains	20015870
Fntb, farnesyltransferase, CAAX box, beta [110606]	2.5x down	N	posttranslational modification of proteins carrying a C-terminal CaaX motif, beta subunit is responsible for peptide binding	15837621, 20106865

DNA Repair

gene symbol, gene name [Entrez gene ID]	EPO modulation, fold change	known / novel	description	reference (PMID #)
Neil3, nei like 3 [234528]	2.3x down	N	DNA glycosylase	20185759
Aen, apoptosis enhancing nuclease [68048]	2.9x up	N	nuclear exonuclease required for p53-dependent apoptosis	18264133
Alkbh1, alkB, alkylation repair homolog 1 [211064]	2.4x up	N	DNA alkylation damage repair by oxidative demethylation, function in placental trophoblast lineage differentiation	18163532
Matr3, matrin 3 [17184]	2.4x up	N	involved in double strand breaks response and repair	20421735

Mitochondrial

gene symbol, gene name [Entrez gene ID]	EPO modulation, fold change	known / novel	description	reference (PMID #)
Atad3a, ATPase family, AAA domain containing 3A [108888]	2.3x up	N	contribute to stabilization of mitochondrial nucleiods	17210950
Timm9, translocase of inner mitochondrial membrane 9 homolog [30056]	2.3x up	N	mitochondrial intermembrane chaperone that mediates the import and insertion of hydrophobic proteins	18022191, 18421298
Chchd4, coiled-coil-helix-coiled-coil-helix domain containing 4 [72170]	3.5x up	N	required for import and folding of small cysteine-containing proteins in the mitochondrial intermembrane space	20026652
Slc25a32, solute carrier family 25, member 32 [69906]	2.9x up	N	transports folate across the inner membranes of mitochondria	16949250, 16165386
Mthfd2, methylenetetrahydrofolate dehydrogenase (NAD+ dependent), methenyltetrahydrofolate cyclohydrolase [17768]	2.8x up	N	mitochondrial bifunctional enzyme with methylenetetrahydrofolate dehydrogenase and methenyltetrahydrofolate cyclohydrolase activities	18804703, 16150419
Ndufaf4, NADH dehydrogenase (ubiquinone) 1 alpha subcomplex, assembly factor 4 [68493]	2.5x up	N	involved in assembly of mitochondrial NADH:ubiquinone oxidoreductase complex (complex I)	19463981
Hk2, hexokinase 2 [15277]	3.2x up	N	mitochondrial hexokinases catalyze the conversion of glucose into glucose-6-phosphate in glucose metabolism	15636050
Slc25a33, solute carrier family 25, member 33 [70556]	2.4x up	N	transports molecules over the mitochondrial membrane	16949250
Timm10, translocase of inner mitochondrial membrane 10 homolog [30599]	3.0x up	N	mitochondrial intermembrane chaperone that mediates the import and insertion of hydrophobic proteins	18022191, 18421298
Peo1, progressive external ophthalmoplegia 1 [226153]	2.1x up	N	critical for lifetime maintenance of mitochondrial DNA integrity	18039713
Spata5, spermatogenesis associated 5 [57815]	2.0x up	N	involved in morphological and functional mitochondrial transformations during spermatogenesis	16465403

Amino Acid Polyamine

gene symbol, gene name [Entrez gene ID]	EPO modulation, fold change	known / novel	description	reference (PMID #)
Srm, spermidine synthase [20810]	3.9x up	N	catalyzes final step of polyamine spermidine biosynthesis	16515550
Mtap, methylthioadenosine phosphorylase [66902]	2.7x up	N	plays a major role in polyamine metabolism and is important for the salvage of both adenine and methionine	19567676
Pcsk4, proprotein convertase subtilisin/kexin type 4 [18551]	2.0x up	N	involved in the processing of hormone and other protein precursors at sites comprised of pairs of basic amino acid residues	16371590, 9482861
Eif1ay, eukaryotic translation initiation factor 1A, X-linked [66235]	3.7x up	N	stabilizes the binding of the initiator Met-tRNA(I) to 40 S ribosomal subunits	11416183

Transporters

gene symbol, gene name [Entrez gene ID]	EPO modulation, fold change	known / novel	description	reference (PMID #)
Slc19a1, solute carrier family 19 (sodium/hydrogen exchanger), member 1 [20509]	2.8x up	N	transporter for the intake of folate	11600421
Slc40a1, solute carrier family 40 (iron-regulated transporter), member 1 [53945]	4.7x down	N	cell membrane protein that may be involved in iron export from duodenal epithelial cells	15084469, 16330432
Slc7a1, solute carrier family 7 (cationic amino acid transporter, y+ system), member 1 [11987]	3.6x up	N	high-affinity, low capacity permease involved in the transport of the cationic amino acids in non-hepatic tissues	14770310, 19485892
Slc16a6, solute carrier family 16 (monocarboxylic acid transporters), member 6 [104681]	2.2x up	N	proton-linked monocarboxylate transporter	16133915, 18471784

Protein Transport Trafficking

gene symbol, gene name [Entrez gene ID]	EPO modulation, fold change	known / novel	description	reference (PMID #)
Rab43, RAB43, member RAS oncogene family [69834]	2.7x up	N	key role in ER-Golgi trafficking	18664496, 17684057
