# Supplementary material for: Defining an EPOR- Regulated Transcriptome for Primary Progenitors, including Tnfr-sf13c as a Novel Mediator of EPO- Dependent Erythroblast Formation
Source: PLoS One. 2012 Jul 13;7(7):e38530. doi: 10.1371/journal.pone.0038530 (PMC3396641; doi:10.1371/journal.pone.0038530)
Supplement: Table S5 — Epo/Epor Modulated Cancer Biology. (PDF) [file pone.0038530.s009.pdf]

SUPPLEMENTAL TABLE S5: EPO/EPOR MODULATED CANCER BIOLOGY

gene symbol, gene name [Entrez gene ID]	EPO modulation, fold change	known / novel	description	reference (PMID #)
Mtdh, metadherin [67154]	2.0x up	N	Promotes lung metastasis and also has an effect on bone and brain metastasis, possibly by enhancing the seeding of tumor cells to the target organ endothelium. Induces chemoresistance	19723648
Dnajb4, DnaJ (Hsp40) homolog subfamily B, member 41 [67035]	2.3x down	N	Activates JNK/JunD pathway; inhibits lung cancer cell invasion and metastasis by modulating E-Cadherin; tumor suppressor	18794131; 20145123
Pvt1, plasmacytoma variant translocation 1 [19296]	3.2x up	N	An oncogene; a downstream target of the c-Myc gene; involved in translocation in variant Burkitt's lymphonas and murine pasmacytomas upregulated in Hidkin's Lymphoma	17503467; 21037568
Cdr2, cerebellar degeneration-related 2 [12585]	2.6x down	N	A tumor antigen; it is cell cycle regulated (peaking in mitosis), it triggers autoimmune response	20383333; 19581925
Tspan14, tetraspanin 14 [52588]	3.5x up	N	Genetically altered in lung cancer; may promote tumor growth	19473719
Ccdc68, coiled-coil domain containing 68 [381175]	2.5x down	N	A tumor supressor; downregulated in primary colon cancer (may be a selective advantage for colon cancer)	19359472
