# Supplementary material for: Defining an EPOR- Regulated Transcriptome for Primary Progenitors, including Tnfr-sf13c as a Novel Mediator of EPO- Dependent Erythroblast Formation
Source: PLoS One. 2012 Jul 13;7(7):e38530. doi: 10.1371/journal.pone.0038530 (PMC3396641; doi:10.1371/journal.pone.0038530)
Supplement: Table S6 — Epo/Epor Modulated Ribosome Biosynthesis. (PDF) [file pone.0038530.s010.pdf]

SUPPLEMENTAL TABLE S6: EPO/EPOR MODULATED RIBOSOME BIOSYNTHESIS

gene symbol, gene name [Entrez gene ID]	EPO modulation, fold change	known / novel	description	reference (PMID or MGI)
Bxdc2, BRX1 biogenesis of ribosomes homolog [67832]	2.1x up	N	60S subunit, ribosomal biosynthesis	12702244
Mak16p, MAK16 homolog [67920]	2.1x up	N	stabilize 27SB precursor rRNA and maturation of 25S and 5.8S rRNA	16710831
Rrs1, RRS1 ribosome biogenesis regulator homolog [59014]	3.7x up	N	export of 60S subunits from nucleolus to cytoplasm	15135061
Bysl, bystin-like [53414]	2.4x up	N	40S ribosome biogenesis	17242206
Nmd3, NMD3 homolog [97112]	2.3x up	N	nucleocytoplasmic shuttling protein, export 60S ribsomal subunit	20584915
Dimt1l, DIM1 dimethyladenosine transferase 1-like [66254]	2.2x up	N	dimethylates adensosines in 18SrRNA	MGI:1913504
Tsr1, TSR1 20S rRNA accumulation homolog [104662]	3.5x up	N	required for maturation og 40S ribsomal subunit	16159874
Rps24, ribosomal protein S24 [677113]	2.2x up	K	nucleotide binding, structural constituent of ribosome, structural constituent of ribosome	1840675
Urb2, URB2 ribosome biogenesis 2 homolog, [382038]	2.2x up	N	molecular functions unknown	MGI:2681124
Rrp1b, ribosomal RNA processing 1 homolog B [72462]	2.9x up	N	protein binding, dynamic modulator of transcription and chromatin structure	18081427, 20040599
Rpl12, ribosomal protein L12 [269261]	4.9x up	N	bind 28S ribosomal RNA	9013569
