# Supplementary material for: Defining an EPOR- Regulated Transcriptome for Primary Progenitors, including Tnfr-sf13c as a Novel Mediator of EPO- Dependent Erythroblast Formation
Source: PLoS One. 2012 Jul 13;7(7):e38530. doi: 10.1371/journal.pone.0038530 (PMC3396641; doi:10.1371/journal.pone.0038530)
Supplement: Table S7 — Epo/Epor Modulated Rna Processing. (PDF) [file pone.0038530.s011.pdf]

SUPPLEMENTAL TABLE S7: EPO/EPOR MODULATED RNA PROCESSING

gene symbol, gene name [Entrez gene ID]	EPO modulation, fold change	known / novel	description	reference (PMID or MGI)
Clk1, CDC-like kinase 1 [12747]	2x down	N	nucleotide binding, protein serine/threonine kinase, tyrosine kinase activity, ATP binding, transferase activity	1825055, 9307018
Polr1b, polymerase (RNA) I polypeptide B [20017]	2.5x up	N	DNA binding, DNA-directed RNA polymerase activity, protein binding, zinc ion binding, nucleotidyltransferase activity, ribonucleoside binding.	9236775, 18023416
Heatr1, HEAT repeat containing 1 [217995]	2.2x up	N	binding	MGI:2442524
Nip7, nuclear import 7 homolog [66164]	2.3x up	N	RNA binding, protein binding, ribosome biogenesis, accurate pre-rRNA processing	16128814, 9891085
Trmt61A, tRNA methyltransferase 61 homolog A [328162]	2.8x up	N	protein binding, tRNA (adenine-N1-)-methyltransferase activity	16043508
Yrdc, yrdC domain containing, [230734]	2.8x up	N	protein binding, needed for the maturation of 16S rRNA	2675563, 15716138
Exosc1, exosome component 1[66583]	2.3x up	N	RNA binding, nuclease activity, exonuclease activity, protein binding, hydrolase activity	11812149, 17174896
