# Supplementary material for: Defining an EPOR- Regulated Transcriptome for Primary Progenitors, including Tnfr-sf13c as a Novel Mediator of EPO- Dependent Erythroblast Formation
Source: PLoS One. 2012 Jul 13;7(7):e38530. doi: 10.1371/journal.pone.0038530 (PMC3396641; doi:10.1371/journal.pone.0038530)
Supplement: Table S8 — Epo/Epor Modulated Other. (PDF) [file pone.0038530.s012.pdf]

SUPPLEMENTAL TABLE S8: EPO/EPOR MODULATED OTHER

gene symbol, gene name [Entrez gene ID]	EPO modulation, fold change	known / novel	description	reference (PMID or MGI)
Rnf141, ring finger protein 141 [67150]	2.2x down	N	plays an important role in mouse spermatogenesis, including spermatogenic cell proliferation and sperm mutation, as well as motility and fertilization	18823591
493042210Rik, RIKEN cDNA 4930422I07 gene [71640]	2.4x down	N	zinc finger protein 949	MGI:1918890
1190002F15Rik, RIKEN cDNA 1190002F15 gene [381822]	2.1x down	N	unknown	MGI:1915081
545228, Gm5817 predicted gene 5817 [545228]	2.2x down	N	unknown	MGI:3779527
AA408296, expressed sequence AA408296 [215193]	2.0x up	N	digestive organ expansion factor homolog (zebrafish)	MGI:2138080
Pcgf3, polycomb group ring finger 3 [69587]	2.1x up	N	unknown	MGI:1916837
LOC636713 or *Srfbp1*, similar to SRF-dependent transcription regulation associated protein serum response factor binding protein 1 [636713/67222]	2.1x up	N	May be involved in regulating transcriptional activation of cardiac genes during aging process; may play a role in biosynthesis and/or processing of SLC2A4 in adipose cells	MGI:1914472
1700097N02Rik, RIKEN cDNA 1700097N02 gene [67522]	2.1x up	N	unknown	MGI:1914772
2310043N10Rik or *Neat1*, nuclear paraspeckle assembly transcript 1 [66961]	2.1x down	N	Plays a role in paraspeckle formation	20211624; 19217333
6230427J02Rik or *Inka1*, RIKEN cDNA 6230427J02 gene [68176]	2.1x up	N	Known as Inka1; gene regulated in the neural crest required for craniofacioal morphogenesis in fish and frog	20175189
D16Ertd472e, DNA segment, Chr 16, ERATO Doi 472 [67102]	2.6x up	N	unknown	MGI:1196400
Snhg7, small nucleolar RNA host gene (non-protein coding) 7 [72091]	2.6x up	N	unknown	MGI:1919341
2810474O19Rik, RIKEN cDNA 2810474O19 gene [67246]	2.6x up	N	unknown	MGI:1914496
Tmem185b, transmembrane protein 185B [226351]	2.6x up	N	Transmembrane protein involved with G-protein-coupled receptors	15525354
Bend3, BEN domain containing 3 [331623]	2.5x up	N	unknown	MGI:2677212
Kbtbd8, kelch repeat and BTB (POZ) domain containing 3 [243574]	2.5x up	N	unknown	MGI:2661430
Gtlf3a, Gm16516 predicted gene, Gm16516 [24082]	3.9x up	K	Expressed during formation of early hematopoietic cells	10402672
Ifi35, interferon-induced protein 35 [70110]	2.3x up	N	interferon-induced leucine zipper protein that undergoes interferon-regulated cellular redistribution	8288566
2410016O06Rik, RIKEN cDNA 2410016O06 gene [71952]	3.2x up	N	Histone demethylase involved in chromatin remodeling	14742713
2700023E23Rik, RIKEN cDNA 2700023E23 gene [70036]	2.9x up	N	unknown	MGI:1917286
Fhl1, four and a half LIM domains 1 [14199]	2.2x up	N	May have an involvement in muscle development or hypertrophy	21310615
Gdap10, Gdap10 ganglioside-induced differentiation-associated-protein 10 [14546]	2.2x up	N	May be involved in the signal transduction pathyway that is triggered through the expresion of a single sialyltransferase gene	10217254
Ccdc137, coiled-coil domain containing 137 [Ccdc137]	2.2x up	N	unknown	MGI:1914541
2810474O19Rik, RIKEN cDNA 2810474O19 gene [67246]	3.1x up	N	unknown	MGI:1914496
Wdr43, WD repeat domain 43 [72515]	2.4x up	N	unknown	MGI:1919765
Nol9, nucleolar protein 9 [74035]	2.4x up	N	unknown	MGI:1921285
Sssca1, Sjogren's syndrome/scleroderma autoantigen 1 homolog [56390]	2.5x up	N	Might play a role in mitosis. Antigenic molecule. Could be a centromere-associated protein. May induce anti-centromere antibodies.	9486406
Fam46c, family with sequence similarity 46, member C [74645]	2.5x up	N	unknown	MGI:1921895
Cnbp, cellular nucleic acid binding protein [7555]	2.8x up	N	May be involved in controlling cell death and proliferation rates	20960530
Ydjc, YdjC homolog [69101]	3.5x up	N	Contains a structural relationship with peptidoglycan N-acetylglucosamine deacetylase	18177738
Sri, sorcin [109552]	2.9x down	N	Calcium-binding protein that modulates excitation-contraction coupling in the heart.	17699613
9530029O12Rik, RIKEN cDNA 9530029O12 gene [399629]	2.3x up	N	unknown	MGI:3026981
Cdv3, carnitine deficiency-associated gene expressed in ventricle 3 [321022]	2.3x up	N	May be involved in the development of cardiac hypertrophy caused by carnitine deficiency	12359334
Nrp, neuropilin 1 [654309]	2.2x up	K	May negatively regulates erythroid stem cell proliferation; an antagonism to IL-3 stimulation	2205877
Ssbp3, single-stranded DNA binding protein 3 [23648]	2.1x down	N	unknown	MGI:1919725
