# Supplementary material for: Defining an EPOR- Regulated Transcriptome for Primary Progenitors, including Tnfr-sf13c as a Novel Mediator of EPO- Dependent Erythroblast Formation
Source: PLoS One. 2012 Jul 13;7(7):e38530. doi: 10.1371/journal.pone.0038530 (PMC3396641; doi:10.1371/journal.pone.0038530)
Supplement: Table S9 — Candidate Regulators Of Tnfrsf13c Expression. (PDF) [file pone.0038530.s013.pdf]

SUPPLEMENTAL TABLE S9: CANDIDATE REGULATORS OF *Tnfrsf13c* EXPRESSION.
Fold-modulation, and relative expression values were estimated from NCBI GEO database profiles (GDS #'s).

**variable**	**fold-modulation**	***control mean +/- SE**	***experimental mean +/- SE**	**tissue/cell type**	**NCBI GEO index #**
IL4	4.3	20.2 +/- 2.9	87.8 +/- 7.8	C57BL/6 B cells	GDS307[a]
IL2	2.0	48.4 +/- 1.6	96.9 +/- 3.1	NK cells	GDS3191[b]
SDF1	3.0	30.2 +/- 5.2	89.9 +/- 8.3	C57BL/6 B cells	GDS318[c]
HSC vs myeloid cells	12.9	6.4 +/- 0.5	82.8 +/- 9.1	hematopoietic	GDS2720[d]
Stat5b knockout	3.3	53.7 +/- 13.7	16.4 +/- 3.6	intestinal	GDS3385[e]
ZFP36L2 deletion	1.6	64.9 +/- 16.3	40.8 +/- 9.2	d14.5 fetal liver	GDS3574[f]
Pu1 knockdown	4.5	84.1 +/- 15.0	18.6 +/- 7.2	hematopoietic stem cells	GDS2411[g]
N-Myc expression	7.0	12.2 +/- 2.5	85.5 +/- 14.5	lung epithelium	GDS2406[h]

*[normalized to percent maximal value for each data set]

[a]Temporal analysis of IL4-modulated transcripts in primary murine B-cells (C57BL/6); 30 min baseline vs 240 min, 124-exposure (n=3)

[b]Temporal analysis of IL2-modulated transcripts in primary human NK cells resting vs 8h to 24h IL2 exposure (n=1 per time-point analysis)

[c]Temporal analysis of SDF1-modulated transcripts in primary murine B-cells; 30 min baseline vs 240 min SDF-1 exposure (n=3)

[d]Analyses of expressed transcripts in SCA1-pos, Kit-pos, Lin-neg LSK cells vs myeloid progenitors (n=3).

[e]Analyses of effects of STAT5b deletion on transcript expression in primary murine colon cells (n=6 wild-type, n=3 STAT5b-null)

[f]Effects of Zfp36l2 deletion on transcript expression in d14.5 (erythropoietic) murine fetal livers (n=4 wild-type, n=5 Zfp36l2-null)

[g]Effects of Pu.1 knockdown on transcript expression in murine hematopoietic stem cells (n=3 wild-type vs n=3 Pu.1 knockdown)

[h]Effects of transgenic N-myc over-expression on transcript levels in murine lung epithelial cells (embryonic d18.5) (n=2).
